# Third generation cephalosporin-resistant (3GCR) *Escherichia coli* and biocide-tolerant heterotrophic bacteria in irrigation water used in *Capsicum annuum* cultivation areas in Kosovo

**DOI:** 10.1038/s41598-026-42583-z

**Published:** 2026-04-06

**Authors:** Elona Tahiri Vela, Rreze M. Gecaj, Dipen Pulami, Arben Mehmeti, Peter Kämpfer, Stefanie P. Glaeser

**Affiliations:** 1 Institute of Applied Microbiology, Justus-Liebig-University Giessen, Giessen, Germany; 2 Department of Food Technology and Biotechnology, University of Prishtina, Prishtina, Kosovo; 3 Department of Plant Protection, University of Prishtina, Prishtina, Kosovo

**Keywords:** River water pollution, Wastewater, *Capsicum annuum*, Antimicrobial resistance, *Escherichia coli*, Quaternary ammonium compound tolerance, Biotechnology, Environmental sciences, Microbiology, Molecular biology

## Abstract

This systematic study investigated the presence of total and third-generation cephalosporin-resistant (3GCR) *Escherichia coli* as well as total and benzyldimethyldodecylammonium chloride (BAC-C12) tolerant heterotrophic bacteria growing under nutrient-rich conditions at 37 °C, among them potential pathogens, in irrigation water (river and well water) potentially contaminated with wastewater, irrigated soils, and pepper (*Capsicum annuum*) fruits. Samples were collected from five vegetable-cultivating fields in Kosovo. The presence of *E. coli* was confirmed in both investigated rivers, all five examined wells, and in a single soil sample. Furthermore, 3GCR *E. coli* were detected in the two rivers and one well. Genomic fingerprinting revealed a high genetic variability among the cultivated *E. coli* strains. The 3GCR *E. coli* strains contained either *bla*_CTX−M_ and *bla*_TEM_ extended spectrum beta lactamase (ESBL) genes and/or exhibited an aminopenicillin-inactivating cephalosporinase (AmpC) phenotype. In addition, certain strains displayed resistance to ciprofloxacin and colistin, although none demonstrated resistance to carbapenems. Beyond (3GCR) *E. coli*, BAC-C12-tolerant bacteria were detected in river and well water, agricultural soil, and pepper fruit samples across all five investigated sites; among them bacteria of the genera *Providencia*, *Morganella*, and *Pseudomonas.* River water as a source of irrigation showed higher levels of microbial contamination compared to well water; however, well water also demonstrated contamination with fecal indicator bacteria. Despite this, no contamination of pepper fruits with fecal indicator bacteria was observed. Nonetheless, the presence of *Pseudomonas* with a high BAC-C12 tolerance at or in *C. annuum* fruits requires a more detailed examination.

**Supplementary Information:**

The online version contains supplementary material available at 10.1038/s41598-026-42583-z.

## Introduction

Untreated wastewater is a highly hazardous source of contamination for aquatic ecosystems due to its high levels of nutrients, organic matter, and pollutants^1,2^. Its discharge into water bodies is a growing global concern that has potential impacts on both public health and the environment, especially in developing countries with insufficient wastewater treatment systems or low application rates^2–4^. A study by Rogowska et al.^5^ emphasizes that these countries have made a paradigm shift by implementing improved nutrient removal techniques in wastewater treatment plants to meet legal effluent discharge limits for biochemical oxygen demand (BOD), chemical oxygen demand (COD), suspended solids, nitrogen (N), phosphorus (P) loads, and other pollutants.

As a newly established country seeking EU integration, Kosovo has aligned its national water legislation with the EU Water Framework Directive^6^. Despite this alignment, Kosovo faces significant shortcomings in wastewater system management, where wastewater from urban, industrial, and agricultural sources is discharged into aquatic environments without prior treatment^7,8^. This environmental issue poses a serious threat to groundwater and surface water quality in Kosovo^9^, with an estimated 74–90% of water sources containing wastewater and fecal pollution^10^. The current research regarding waters^11,12^, sediments, and surrounding soils^13,14^ in Kosovo is scarce, and has primarily focused on the chemical quality. Shala et al.^15^ reported water contamination in Kosovo with high concentrations of metal ions (Fe, Mn, Zn, Ni) in irrigation water near rivers, frequently exceeding the EU Standards for metal ions of 198 mg L^− 1^^16^. In contrast, biological aspects are still in the early stages of assessment^17,18^. The use of untreated wastewater-contaminated water for agricultural purposes can introduce bacterial contaminants (pathogenic and antibiotic-resistant bacteria, ARB) to soil and plants, posing the risk of transmission of waterborne and foodborne diseases to humans through the consumption of contaminated raw food^19–21^.

Antibiotic resistance is spreading at an alarming rate worldwide due to the misuse and overuse of antibiotics becoming a global challenge^22–25^. It is estimated that by 2050 there will be approximately 10 million deaths/year on account of issues that are related to antibiotic resistance^26^. Nearly every class of antibiotics, ARB, and antibiotic resistance genes (ARGs) have been detected in wastewater and various environmental samples, including river water and sediments, groundwater, as well as drinking water^22,27,28^.

In addition, the environmental contamination with biocidal compounds such as quaternary ammonium compounds (QACs) and bacteria tolerant to these compounds, are receiving increasing attention. Biocidal compounds (e.g., QACs) and heavy metals, can induce co-selection of antimicrobial resistances even at sublethal concentrations due to co- and cross-resistance mechanisms^29–33^.

As a developing country, Kosovo faces significant challenges in monitoring antimicrobial resistance (AMR), and resistance rates are reported to be two to five times higher than EU standards. Key contributors to these elevated rates include the over-the-counter sale of antimicrobials in pharmacies, pressure from the pharmaceutical industry to prescribe specific antibiotics, the lack of officially approved antibiotic guidelines in hospitals, limited access to point-of-care diagnostics in primary care, shortcomings in the documentation process, and the underutilization of medical microbiological diagnostics^34^. Ibraimi et al.^35^ investigated approximately 80% of the dairy cattle farms in Kosovo and found general problems by the correct application of antibiotics, which are mainly beta-lactams and sulfonamides. Less is known about the spread of AMR in the environment in Kosovo. To the best of our knowledge there is only one comprehensive report on AMR in *E. coli* and enterococci which were isolated from dairy farms across a broad geographical area of Kosovo^36^. The authors reported several priority resistance phenotypes among the cultivated bacteria including resistance to cephems, quinolones, and macrolides.

To address the significant gap in understanding the dynamics of AMR transmission through potentially wastewater-contaminated irrigation waters into the food chain in Kosovo, we conducted our study based on the following hypotheses: (i) The discharge of untreated wastewater into aquatic environments leads to the transmission of potentially pathogenic and ARB into the food chain via the use of wastewater-contaminated irrigation water from river and well sources. (ii) Increased use of disinfectants since the COVID-19 pandemic, with a peak after the onset of the pandemic, may have contributed to a high prevalence of bacteria that are tolerant to biocidal compounds such as QACs in wastewater and the receiving environment. (iii) River water is expected to contain higher concentrations of ARB and bacteria tolerant to biocidal compounds than well water and may result in a higher contamination rate of pepper fruits. To test these hypotheses, the environmental spread of *E. coli* as indicator for fecal pollutions, 3GCR *E. coli*^37^ as ARB indicator, and heterotrophic bacteria culturable under high nutrient conditions at 37 °C with tolerance to the biocidal compound BAC-C12, was studied. This QAC was selected because it is amongst the most prominent QAC homologue in many disinfectant agents^32,38–40^ and was amongst the most abundant QAC homologues detected for example in suspended organic matter of German river samples^41^.

**Fig. 1:**
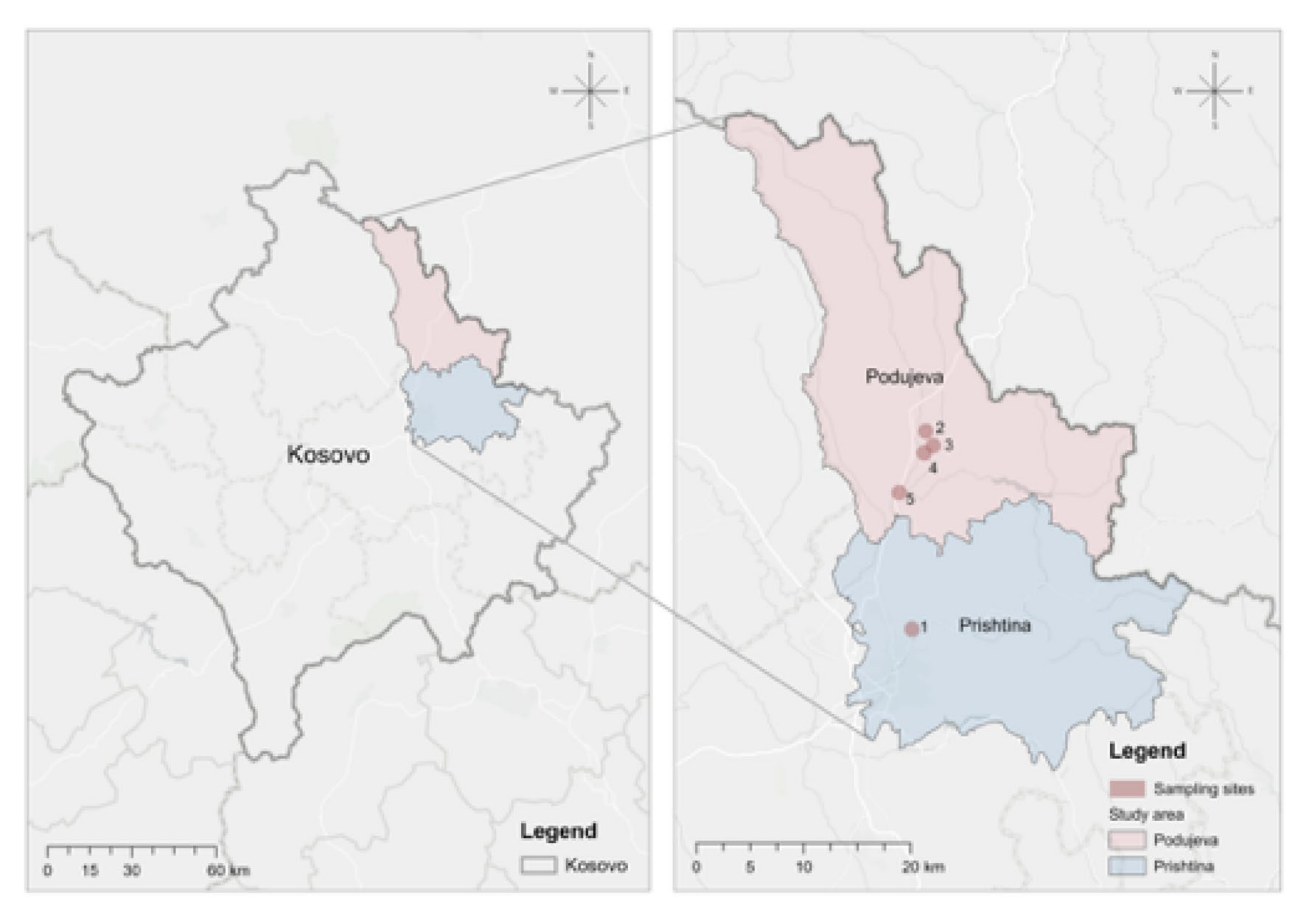
Overview of the five sampling locations in Kosovo

Irrigation water (groundwater from on-farm wells and surface water from local rivers), topsoil from agricultural fields planted with *Capsicum annuum* (pepper) (Supplementary Fig. S1), and the raw bell pepper fruits at the time of harvest were collected at five geographically different vegetable-growing areas in Kosovo (locations 1 to 5; Fig. 1) for cultivation-dependent analysis.

Two cultivation strategies were applied: quantitative direct plating (DP) and qualitative non-selective pre-enrichment (PE) cultivation to enhance the cultivability of the selected target bacteria.

## Results

### *E. coli* and 3GCR *E. coli* cultivated from river and well water and selected soil samples but not from pepper fruits

**Fig. 2:**
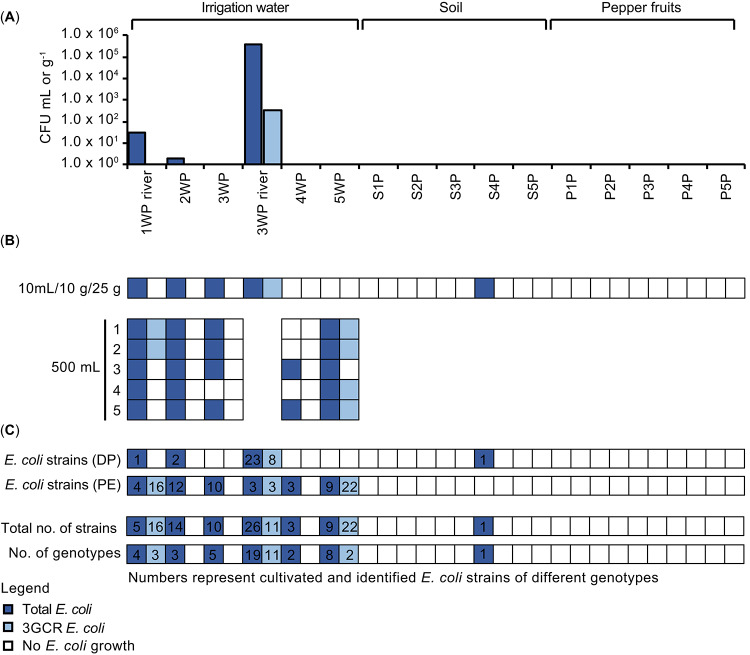
*E. coli* and 3GCR *E. coli* cultivated from irrigation water, soil, and pepper fruits. (**A**) Quantitative detection of *E. coli* and 3GCR *E. coli* by direct plating (DP) cultivation. The results are presented as colony-forming units (CFUs) per mL of water, per g dry weight of soil, or per g fresh weight of pepper fruits. (**B**) Qualitative detection of *E. coli* and 3GCR *E. coli* following non-selective pre-enrichment (PE). Given amounts (mL, g) represent the analysed amount of irrigation water, soil, and pepper sample used for PE. (**C**) Overview of the confirmed cultivated *E. coli* and 3GCR *E. coli* strains and number of different *E. coli* genotypes (defined based on BOX-PCR fingerprint patterns). Dark blue boxes: *E. coli*; light blue boxes: 3GCR *E. coli*. Sample IDs: 1–5W, S1-S5, and P1-P5: water (well water samples unless otherwise indicated as river water), topsoil, and pepper fruit samples from locations 1 to 5

*Escherichia coli* was detected by DP cultivation in water samples of the two rivers collected at locations 1 and 3 and of well water collected at location 2 (Fig. 2A). The concentrations were in the range of 10^1^, 10^5^, and 10^0^ CFU mL^− 1^, respectively. Quantitative detection of 3GCR *E. coli*, was only possible in the river water sample of location 3, with a concentration in the range of 10^2^ CFU mL^− 1^.

Pre-enrichment based cultivation enabled the detection of *E. coli* in both rivers and all well water samples (Fig. 2B). However, 3GCR *E. coli* were only cultivated from the two rivers and one well water sample. Neither *E. coli* nor 3GCR *E. coli* were detected by DP from soil and pepper fruit samples (Fig. 2A). *E. coli* was only detected in one soil sample after PE, but not in any of the pepper fruits samples, even after PE cultivation. 3GCR *E. coli* were not detected by PE cultivation in soil or pepper fruits. When comparing the number of positive sample replicates obtained by PE from the less contaminated water sources (river at location 1 and other well water samples), the well water from location 4 showed the lowest detection rate of *E. coli* contamination, with only two of five 500-mL samples testing positive for *E. coli* and none for 3GCR *E. coli*. In contrast, although no *E. coli* were detected in well water from location 5 by either DP or PE cultivation from a 10-mL sample, the 500-mL samples from this well exhibited the highest contamination rate after PE, all five replicates were positive for total *E. coli*, and four were positive for 3GCR *E. coli* (Fig. 2B).

### Genotypic characterization of the cultivated *E. coli* and 3GCR *E. coli* strains

A total of 68 *E. coli* strains cultivated on tryptone bile X-glucuronide (TBX) without CTX were preserved and further characterized (Fig. 2C). All strains showed an *E. coli* phenotype on TBX agar and tested positive for the presence of the *E. coli* typical glutamate decarboxylase (GAD) genes *gadA/B*^42^. The *E. coli* strains represented 42 genotypes (defined by genomic fingerprinting using BOX-PCR patterns, Fig. 2C). The highest genetic variability was obtained for river water of location 3 (19 genotypes), while for river water of location 1 only four genotypes were obtained. The well water of location 5 showed the second highest genetic variability of *E. coli* strains (eight different genotypes). In comparison, five genotypes were cultivated from well water at location 3 and three and two from well water at location 2 and 4. The strain cultivated from the soil sample of location 4 showed a unique genotype.

**Fig. 3:**
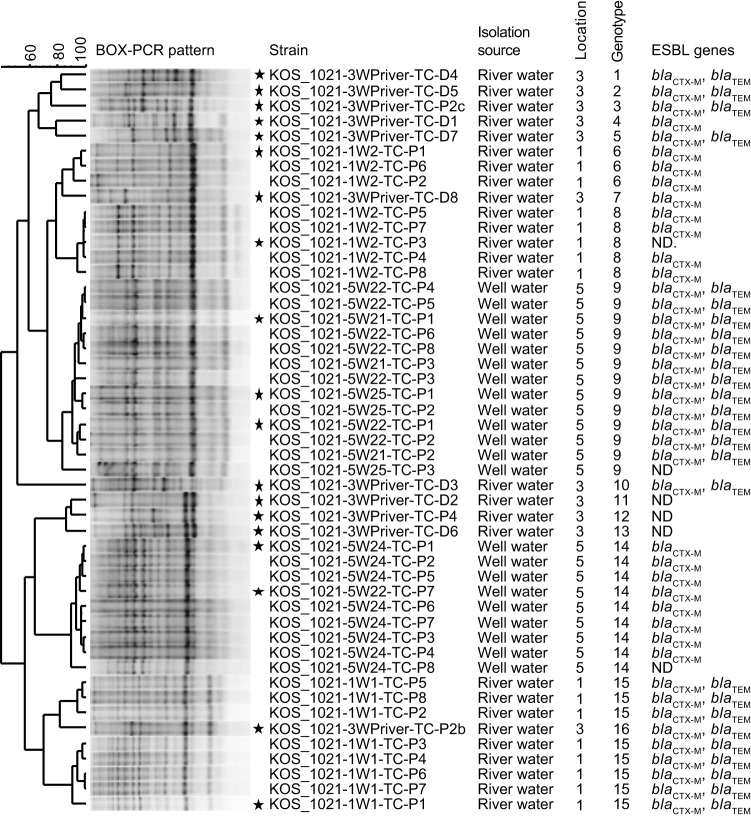
Overview of the genetic diversity of the cultivated 3GCR *E. coli* strains

A total of 49 3GCR *E. coli* strains were cultivated from river water (location 1 and 3) and well water (location 5) (Fig. 2C). Genomic fingerprinting revealed 16 different genotypes (Fig. 3). The 3GCR *E. coli* strains cultivated from different locations represented different genotypes. The genotypes of the 3GCR *E. coli* strains were also all different to the genotypes obtained for *E. coli* strains which were cultivated under non-selective cultivation conditions (TBX without CTX). The river water of location 3 displayed the highest genotype diversity of 3GCR *E. coli* strains (11 genotypes); three different genotypes were cultivated from river water of location 1 and two from well water of location 5. Among the 49 3GCR *E. coli* strains, 26 carried both, *bla*_CTX−M_ and *bla*_TEM_ genes, 17 strains only a *bla*_CTX−M_ gene, but none carried a *bla*_SHV_ gene. Six strains did not carry any of the tested ESBL genes (Fig. 3).

### Antibiotic susceptibility and QAC tolerance testing of cultivated 3GCR *E. coli*

**Fig. 4:**
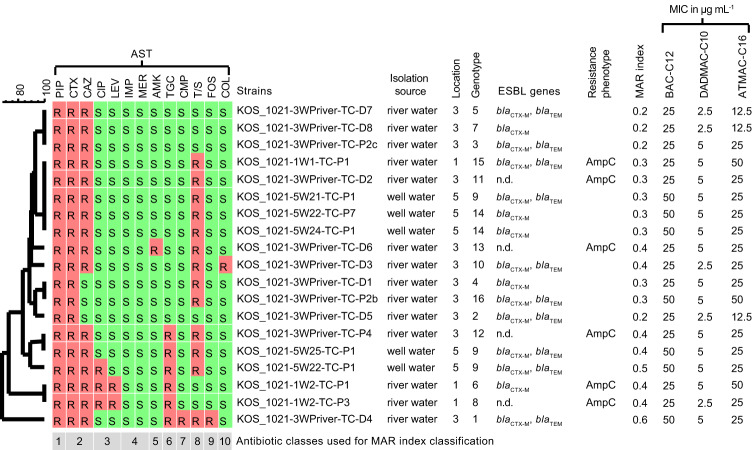
Antibiotic susceptibility profiles and QAC tolerance of selected 3GCR *E. coli* strains. Dendrogram based on UPGMA clustering based on resistance patterns obtained by AST (BioNumerics). PIP, Piperacillin; CTX, cefotaxime; CAZ, ceftazidime; CIP, ciprofloxacin; LEV, levofloxacin; IMP, imipenem; MER, meropenem; AMK, amikacin; TGC, tigecycline; CMP, chloramphenicol; T/S, trimethoprim/sulfamethoxazole; FOS, fosfomycin; COL, colistin; MAR, multiple antibiotic resistance index based on the number of antibiotic classes with resistances as a fraction of the total number of tested classes; MIC, Minimum inhibitory concentration; BAC-C12, Benzyldimethyldodecylammonium chloride; DADMAC-C10, didecyldimethylammonium chloride; ATMAC-C16, hexadecyl trimethyl ammonium chloride. S: Susceptible; R, resistant. Explanation to strain names in legend of Fig. 3

Nineteen 3GCR *E. coli* strains, including all different genotypes, were selected for AST. All strains were resistant to piperacillin and cefotaxime (Fig. 4). Sixteen of the strains were also resistant to ceftazidime. Most of the strains were susceptible to ciprofloxacin (16 out of 19) and levofloxacin (17 out of 19); only three strains were resistant, two from river water of location 1 and one from well water of location 5. All strains were susceptible to imipenem and meropenem. Only single strains cultivated from water samples of river 3 were resistant to either amikacin, chloramphenicol, fosfomycin, or colistin. Six strains were resistant to tigecycline and 13 to trimethoprim/sulfamethoxazole. Four of the 19 strains (21%) had a multiple antibiotic resistance (MAR) index of 0.2 and 15 strains (79%) had a MAR index of 0.3 to 0.6. The 3GCR *E. coli* strains, where none of the tested ESBL genes was detected, exhibited an AmpC phenotype. The minimal inhibitory concentration (MIC) values for the tested QACs were in the range of 25 to 50 µg BAC-C12 mL^− 1^, 2.5 to 5 µg DADMAC-C10 mL^− 1^, and 12.5 to 50 µg ATMAC-C16 mL^− 1^, respectively.

### Detection of other non-target bacteria during (3GCR) *E. coli* cultivation in water, soil, and pepper fruits

**Fig. 5:**
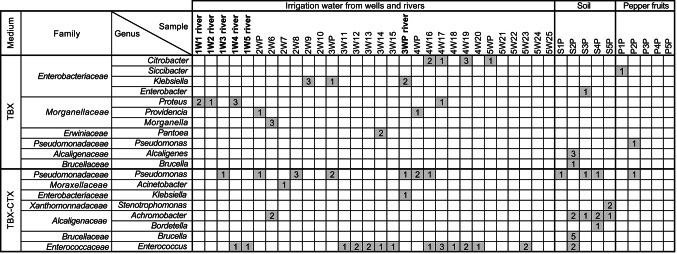
Overview of phylogenetically identified cultivated non-target bacteria grown as beige colonies on TBX and TBX agar supplemented with 1 µg CTX mL^− 1^ (TBX-CTX). Numbers in boxes represent the number of individual strains cultivated from the individual samples. First numbers in sample names represent the respective locations (1 to 5), numbers behind the sample type represent subsamples analyzed (either individual replicate numbers or “P” for pooled samples). W: water, S: soil, and P: pepper fruit. For a more detailed phylogenetic placement see Fig. 6

**Fig. 6:**
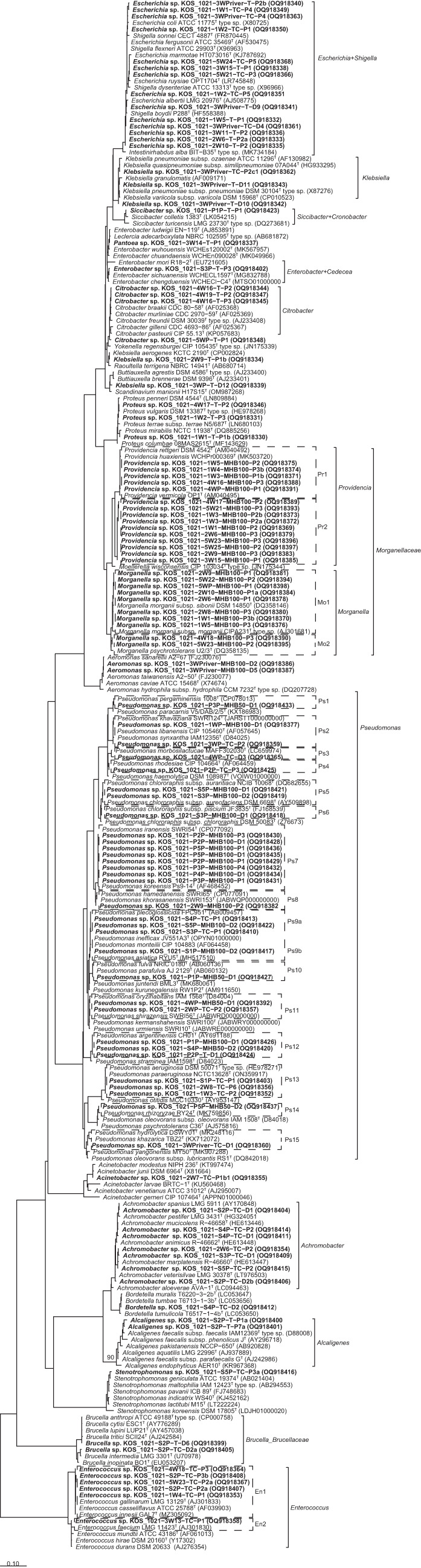
Phylogenetic placement of all cultivated bacterial strains in a 16S rRNA gene sequence-based tree. Partial sequences (around 1000 nt) were added to the LTP database using ARB. A reduced database subtree including the strains cultivated in this study is depicted. The tree contains only sequenced representatives of different cultivated bacterial genotypes

Beside colonies with an *E. coli* phenotype, 88 beige-pigmented colonies (34 from TBX agar and 54 from TBX agar supplemented with 1 µg cefotaxime mL^− 1^ (TBX-CTX) were selected randomly for further analysis from water, soil, and pepper fruit samples (Fig. 5). Strains cultivated on TBX agar were assigned to *Citrobacter*, *Klebsiella*, *Proteus*, *Providencia*, *Morganella*, and *Pantoea* (river and/or well water), *Enterobacter*, *Alcaligenes*, and *Brucella* (soil), and *Siccibacter* and *Pseudomonas* (pepper fruits). The *Klebsiella* strains showed highest 16S rRNA gene sequence identity (> 99.9%) to the type strain of *Klebsiella pneumoniae* (Fig. 6).

Strains cultivated on TBX-CTX agar represented *Pseudomonas* (both water types, soil, pepper fruits), *Enterococcus* (river and well water, soil), *Achromobacter* (well water, soil), *Klebsiella* (river water), *Acinetobacter* (well water), and *Stenotrophomonas*, *Brucella*, and *Bordetella* (soil). For most taxa, high diversity was observed, including several phylotypes (Fig. 6) and genotypes (data not shown). Five *Pseudomonas* strains (well water , soil) isolated from TBX-CTX shared highest 16S rRNA gene sequence similarity (100%) to the type strain of *P. aeruginosa* (phylotype Ps13, Fig. 6). These strains, isolated from well water and soil, represented three different genotypes (Supplementary Fig. S2). Only representatives of genetically different strains (one representative per genotype) were selected for 16S rRNA gene sequencing. *Enterococcus* strains isolated from TBX-CTX were assigned to two phylotypes (Fig. 6). Phylotype E1 was represented by 11 strains from water and soil, which shared highest 16S rRNA gene sequence identity to type strains of *Enterococcus gallinarum* and *E. casseliflavus* (99.6%). Genomic fingerprinting showed a slight genetic variability among the 11 strains (Supplementary Fig. S3). Phylotype E2 was represented by ten genotypically identical strains (from three different well water samples) and shared highest 16S rRNA gene sequence similarity to the type of strain of *E. faecium* (> 99.8%).

### Heterotrophic bacteria and BAC-C12 tolerant heterotrophic bacteria cultivated under high nutrient conditions at 37 °C from water, soil, and pepper fruits

**Fig. 7:**
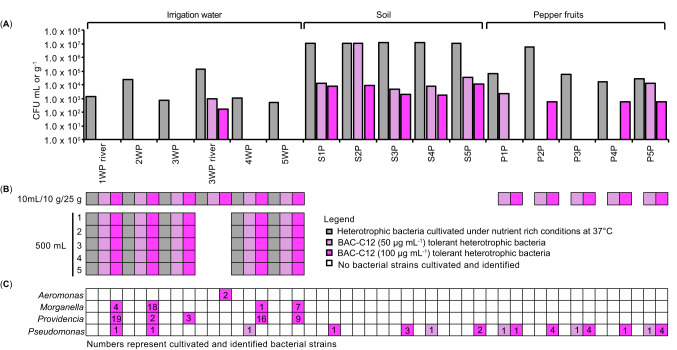
Cultivation of heterotrophic bacteria growing at high nutrient conditions at 37 °C, among them potential pathogens, tolerant to BAC-C12 from water, soil, and pepper fruit samples. Cultivation of total heterotrophic bacteria (MH, 37 °C) and those tolerant to BAC-C12 cultivated on MH with 50 and 100 µg BAC-C12 mL^− 1^. (**A**) Quantitative detection by direct plating (DP). The results are presented as colony-forming units (CFUs) calculated per mL or g (dry weight). (**B**) Qualitative detection after non-selective PE. (**C**) Overview of the taxonomic assignment of cultivated bacterial strains. Phylogenetic identification based on partial 16S rRNA gene sequencing (∼1000 nt) with assignment at the genus level based on at least 98.5% 16S rRNA gene sequence similarities to type strain of next related species (BLAST, NCBI database). Grey, heterotrophic bacteria culturable on MH at 37 °C; light pink, fraction of those bacteria culturable in the presence of 50 µg BAC-C12 mL^− 1^; dark pink: fraction of those bacteria culturable in the presence of 100 µg BAC-C12 mL^− 1^

Heterotrophic bacteria were cultivated on MH agar at 37 °C from all sample types (Fig. 7A). For water samples the concentration was in the range of 10^2^ to 10^5^ CFU mL^− 1^, for soil samples above at least 10^7^ CFU g (dry weight)^−1^ and in pepper fruit samples in the range of 10^4^ to 10^6^ CFU g (dry weight)^−1^.

The cultivation of BAC-C12 tolerant heterotrophic bacteria culturable at high nutrient conditions at 37 °C by DP was successful for river water at location 3 (10^3^ and 10^2^ CFU mL^− 1^ in the presence of 50 and 100 µg BAC-C12 mL^− 1^) and all soil samples (10^3^–10^4^ CFU g (dry weight)^−1^); at location 2 even 10^7^ CFU g (dry weight)^−1^ on MH with 50 µg BAC-C12 mL^− 1^ and 10^3^–10^4^ CFU g (dry weight)^−1^ on MH with 100 µg BAC-C12 mL^− 1^ for all soil samples (Fig. 7A).

For pepper fruits, BAC-C12 tolerant heterotrophic bacteria culturable at high nutrient conditions at 37 °C were cultivated by DP from four out of five pepper samples with inconsistent growth either on MH with 50–100 µg BAC-C12 mL^− 1^. The concentrations were in the range of 10^2^–10^4^ CFUs g (dry weight)^−1^ (Fig. 7A).

Non-selective PE-based cultivation further increased the detection efficiency of BAC-C12 tolerant heterotrophic bacteria in water and pepper fruit samples (Fig. 7B).

One hundred and eight BAC-C12 tolerant bacterial strains were identified by partial 16S rRNA gene sequencing as *Gammaproteobacteria.* Strains of water samples represented *Aeromonas*, *Morganella*, *Providencia*, and *Pseudomonas* (84 strains), strains of soil (seven strains) and pepper (17 strains) were classified as *Pseudomonas* (Fig. 7C). The strains showed a high phylotypic (Fig. 6) and genotypic diversity.

Water-derived BAC-C12 tolerant *Morganella* (30 strains) and *Providencia* (49 strains) were assigned to only two different phylotypes (Fig. 6), with a high genotypic diversity (10 and 30 different genotypes, respectively (Supplementary Fig. S4 and S5). Different genotypes were detected from different water sources. *Pseudomonas* sp. strains isolated in the presence of BAC-C12 represented 11 different phylotypes (Fig. 6) and 21 different genotypes (Supplementary Fig. S2). Most phylotypes were only represented by one or two strains and cultivated from different locations. One phylotype was repeatedly isolated from pepper fruits (Ps7, Fig. 6; Supplementary Fig. S2). Strains of this phylotype (Ps7) shared 100% 16S rRNA gene sequence identity to the type strains of *Pseudomonas iranensis* and *Pseudomonas koreensis* (Fig. 6).

## Discussion

With a cultivation-dependent approach we were able to show that river and well water samples used for irrigation of agricultural fields in Kosovo are contaminated with *E. coli*, 3GCR *E. coli*, and BAC-C12 tolerant heterotrophic bacteria growing at 37°C at high nutrient conditions, among them potential pathogens. The use of a non-selective PE cultivation enhanced clearly the detection efficacy as reported for numerous bacterial pathogens in environmental samples^43–48^.

In this study, a high prevalence for the fecal contamination indicator *E. coli* was identified, particularly in irrigation water. Contamination was observed not only in river water but also in groundwater from deep wells and in soil irrigated with well water. The river water contamination was strongly affected by the sampling location. The main river studied, the Llapi river, which was in proximity to residential housing (location 3), showed higher contamination levels compared to the studied smaller river (location 1) which was located further away from houses. One of the well water samples showed a comparable contamination to that of the smaller river at location 1. Furthermore, cultivation following PE confirmed consistently high levels of *E. coli* contamination in the remaining well water samples across the study sites, supporting high contamination levels in Kosovo. Comparable findings for river water contaminations were reported in Albania, a neighboring country, where rivers near Tirana showed high prevalence of *E. coli* with concentrations exceeding 2 × 10^3^ CFU mL^− 1^ with no significant differences observed in these levels between the wet and dry seasons^49^. In the other neighboring country, Serbia, a critical *E. coli* pollution was shown for the Danube River, especially near Belgrade in the stretch between Novi Sad and Velika Morava^50^. Similar findings were reported by Vinueza et al.^51^ when assessing the microbial contamination of the main rivers in Ecuador, where all rivers exhibited *E. coli* concentrations that exceeded the maximum safety limits set by the United States of America (10^2^ CFU mL^− 1^) in the Recreational Water Quality Criteria and European Union guidelines (10^3^ CFU mL^− 1^). According to the microbiologically based classification system of water quality used by Kirschner et al.^50^, the river water of location 3 showed an excessive fecal contamination (10^7^ CFU/ 100 mL) at the time of sampling and the river water of location 1 showed moderate pollution. Using genomic fingerprinting we were able to estimate the genetic diversity of cultivated *E. coli* at different locations. The *E. coli* diversity in the excessive contaminated river water at location 3 was much higher compared to the sample from the other river and the well water samples.

The European Food Safety Authority has proposed *E. coli* as an indicator for detecting resistances^52^. As a result, the use of antimicrobial-resistant *E. coli* to analyze water quality with respect to ARB to establish its suitability for irrigation is on the horizon. With the applied cultivation methods, we were able to detect 3GCR *E. coli* in our study in both river water samples and in well water of location 5. Many of the strains were identified as ESBL-producing *E. coli* (ESBL *E. coli*). Again, the genetic diversity of 3GCR *E. coli* in the river water samples of location 3 exceeded those of the other river and the well water samples. Our findings are comparable to those of Banu et al.^53^ who reported a significant prevalence of ESBL *E. coli* in river waters from two urban districts in Ghana, Afrika. Banu et al.^53^ reported the presence of ESBL *E. coli* in 98% of the river samples tested which attributed to an increased contamination with ESBL *E. coli* by wastewater discharges into the rivers. On the other hand, a study conducted in Nigeria reported a lower proportion (28%) of ESBL *E. coli* strains in river water samples^54^. A high prevalence of ESBL *E. coli* in surface water used for irrigation in Europe was shown for the major vegetable growing areas of Switzerland^55^. In that study 22% of the tested water samples (eight of 36 samples) were positive for ESBL *E. coli*. All cultivated ESBL *E. coli* strains of that study were multidrug resistant highlighting the problem of AMR transmission in surface waters used for irrigation in Europe.

Although *E. coli* and 3GCR *E. coli* were detected in river and well water samples in our study, *E. coli* were detected in only one soil sample from a field irrigated with well water. A correlation between irrigation water contamination and *E. coli* detection in irrigated soils was not found. *E. coli* transmission to raw pepper fruit samples was also not observed, regardless of the irrigation water contamination. Drip irrigation was applied directly to the plant root zone, thereby improving water-use efficiency compared with conventional furrow irrigation. During soil sampling, a defined radius around each plant root zone was maintained to minimize potential root disturbance, and soil samples were subsequently collected in a randomized manner across the field. This localized and hygienic irrigation practice likely prevented *E. coli* contamination. In contrast several other studies indicate that exposure to contaminated irrigation water led to the contamination of fresh produce with *E. coli*^47,56–59^. Different studies have even shown the contamination of vegetables with Shiga toxin-producing *E. coli* which poses a substantial threat to public health^47,60–62^. Among various contamination pathways for this produce irrigation water is considered as the most prominent pathway^63^. The fact that *E. coli* detection in irrigated soil was negative, even after non-selective PE, does not necessarily mean that *E. coli* were absent. Davidovich et al.^64^ described this as an “under the radar phenomenon” and highlighted the detection problem of pathogens that persist in the environment at concentrations which are below the detection limit.

The majority of the 3GCR *E. coli* strains cultivated from river and well water carried a combination of *bla*_CTX−M_ and *bla*_TEM_ genes. This observation is consistent with previous reports for ESBL *E. coli* from different human impacted environments including surface water samples^45,55,65–67^. Sequence based characterization of *bla*_TEM_ genes showed that the *bla*_TEM_ genes mostly did not represent ESBL genes, but the narrow-spectrum variant TEM-1 that has no ESBL activity^68^. The high abundance of *bla*_CTX−M_ genes is consistent with other studies^55,68–71^. Gekenidis et al.^55^ and Mirkalantari et al.^72^ showed that *bla*_CTX−M_ genes are significantly associated with a MDR phenotype. Few 3GCR *E. coli* strains in our study lacked ESBL genes but showed an AmpC phenotype. Several reports are available that also demonstrated the presence of AmpC producing *E. coli* beside ESBL *E. coli* in irrigation water or fresh produce^57,73^.

Antibiotics of 10 classes were used to evaluate the MDR status of 3GCR *E. coli* by calculating a MAR index. This index was introduced by Krumperman^74^ as an arbitrary value for risk assessment. He considered that bacteria with a MAR index above 0.2 originate from a high-risk contamination site. Kaspar et al.^75^ applied the MAR index concept to water-derived *E. coli* and found a significant difference between *E. coli* of urban and rural rivers. The authors indicated the usefulness of the MAR index to identify high contaminated water sources and to assess the water quality. A study of *E. coli* from river water samples collected from Indian rivers with different wastewater impact, indicated that *E. coli* from river water sites upstream of wastewater discharge were characterized by a MAR index below 0.2 while *E. coli* from mid-stream points had a MAR-index of 0.27 and from up-stream points, high wastewater inflow had a MAR index of 0.38 and larger. The authors concluded from their study that a MAR index above 0.25 can be used to indicate a high-risk contamination of a river. In our study 79% of the 19 tested 3GCR *E. coli* strains had a MAR index of 0.3 and above and 42% of the MAR index of 0.4 and above. This classifies the studied water bodies at least as irrigation water sources with a high contamination risk, especially for the river waters. There were no clear differences among the MAR indices obtained for the 3GCR *E coli* strains from the different water sources or sampling sites in our study. We must consider that we only studied the MAR index of 3GCR *E coli* which have in general higher MAR indexes than non-3GCR *E. coli*^76^. This is mainly caused by the abundance of plasmids in those strains which normally carry more than one resistance gene^77^.

Several of the non-*E. coli* strains cultivated under the high nutrient conditions at 37 °C were assigned to genera which harbor pathogenic species, and even shared high (> 99%) partial 16S rRNA gene sequence similarities to type strains of species which harbor pathogenic strains; among those, *Acinetobacter*,* Alcaligenes*, *Citrobacter*, *Enterobacter*,* Klebsiella* with strains next related to *Klebsiella pneumoniae*, *Morganella*, *Proteus*, *Providencia*, *Pseudomonas* with strains of the species *P. aeruginosa*, and *Stenotrophomonas*. Those taxa were cultivated form water and soil samples which indicated the anthropogenic contamination such as, agricultural runoff or released wastewater from the nearby houses. This is at least valid for location 3 in our study. Among the cultivated non-target bacteria Gram-positive enterococci were also cultivated on TBX agar with CTX from several of the river and well water samples and a soil sample. Enterococci have an intrinsic resistance to cephalosporins which explains their selective cultivation in the presence of CTX^78^. Their high level of intrinsic resistance increases their ability to compete in antibiotic polluted environments against susceptible bacteria. Beside *E. coli*, intestinal enterococci (*E. faecium*, *E. faecalis*, *E. durans*, and *E. hirae*) are the second most prominent indicator for fecal contamination of the environment^79^. Solaiman et al.^80^ also showed the contamination of the different irrigation water types (river water, pond water, and reclaimed water) with both, *E. coli* and enterococci. In our study we determined strains next related to *E. faecium* in several river and well water samples even with a non-enterococci targeting approach. *E. faecium* with vancomycin resistance is listed on the updated WHO bacterial priority pathogens as one of the high-risk pathogens^37^. Ben Said et al.^81^ also determine *E. faecium* as the most prevalent enterococci in irrigation water, soil, and produced vegetable food at farms in Tunisia. A comparative study of food associated and clinical strains of the two clinically relevant species *E. faecium* and *E. faecalis* underlined the clinical importance of food-borne antibiotic-resistant enterococci^82^.

It is widely discussed that the increased use of disinfectants during the SARs-CoV-2 pandemic has affected antimicrobial-resistance spread^32,40,83–85^. Harrison et al.^86^ for example showed that the exposure to increased concentration of BACs had a positive selective effect on ciprofloxacin and sulfamethoxazole resistant bacteria in microcosm experiments with surface water bacterial communities. For those reasons we cultivated selectively QAC tolerant bacteria. We determined a high prevalence of those in river water. Cultivation from well water was just possible after non-selective PE. The cultivated bacteria were assigned to *Providencia*,* Morganella*,* Aeromonas*, and *Pseudomonas.* Only QAC tolerant *Pseudomonas* were also cultivated from irrigated soils and pepper fruits. All cultivated BAC-C12tolerant strains represented genera which contain species that are known to cause infectious diseases to humans and are assigned to risk group 2^87,88^. There are only a few studies available, which also cultivated bacteria in the presence of QACs as selective agents from the environment. Gaze et al.^30^ cultivated a low diversity of QAC tolerant bacteria (*Bacillus*, *Paenibacillus*, and *Pseudomonas)* from agricultural soils but a higher diversity from QAC contaminated read beds including bacteria of the genera *Acinetobacter*, *Aeromonas*, *Citrobacter*, *Enterobacter*, *Serratia*, and *Stenotrophomonas*. Recently, Guo et al.^89^ reported the cultivation of *Lysinibacillus*, *Bacillus*, and *Klebsiella* in the presence of QACs from different soils collected in China. While QAC tolerant *Pseudomonas* and *Aeromonas* were also cultivated in other studies, the high abundance of QAC tolerant *Morganella* and *Providencia* was not reported yet. There is only one early report available showing a high QAC tolerance in *Providencia* strains isolated from wastewater treatment plant effluent and river water in Germany^90^.

Several studies described a broad range of intrinsic QAC tolerance mechanisms of *Pseudomonas*^40^. It was also shown that *Pseudomonas* and *Aeromonas* strains can degrade QACs^91,92^. However, the QAC tolerance of *Aeromonas* spp. is not well studied and even less is known for QAC tolerance in *Morganella* and *Providencia*.

We further evaluated the tolerance of cultivated 3GCR *E. coli* to QAC homologues which are abundant in wastewater, BAC-C12, DADMAC-C10, and ATMAC-C16^32^. The cultivated 3GCR *E. coli* had MIC values to tested QACs which were in the same range as for 3GCR *E. coli* isolated from Germany and Mexico from untreated and treated wastewater or wastewater irrigated plants (unpublished data). Compared to Mexican samples the QAC MIC values were even one dilution higher (data not shown). In *E. coli* QAC resistance genes (mostly coding for efflux pumps) are often co-located with antibiotic resistance genes on plasmids^93^. The presence of QACs can therefore contribute to the transmission of mobile genetic elements and ARGs in the environment. Currently no data are available for environmental QAC contaminations in Kosovo. But we obtained a similar or even higher tolerance of the cultivated *E. coli* strains to QACs as obtained for *E. coli* strains of countries with known high environmental QAC contaminations as Mexico or Germany^2,41,94^. This indicates that also Kosovo may have a high environmental contamination with QACs. Recent studies showed that the abundance of QACs was increased in wastewater in several countries after the COVID-19 pandemic started^95,96^ and Heyde et al.^97^ showed that long-term irrigation with untreated wastewater leads to a strong accumulation of QACs in soils of the Mezquital Valley in Mexico. This aspect must be considered also in Kosovo for a more detailed risk assessment for the use of wastewater contaminated irrigation water. It was already reported that agricultural soils in Kosovo are contaminated with heavy metals^98–100^. Resistance to both, biocidal compounds and heavy metals, can affect AMR transmission in agricultural systems^101,102^. Both, QAC and heavy metal resistance genes, mostly coding for efflux pumps, are often co-located together on plasmids with antimicrobial resistance genes^103^. We repeatedly cultivated one QAC tolerant *Pseudomonas* phylotype (Ps-7) from pepper fruits of three locations. This phylotype was placed into the *P. fluorescens* subgroup *koreensis* which contains at least 22 species^104^ among those *P. iranensis*^105^ and the very closely relative “*P. mercuritolerans*“^106^. For the type strains of “*P. mercuritolerans*“ it was even shown that it can protect forage plants against oxidative stress and supports their growth in the presence of mercury^106^. If there is a linkage between the occurrence of the Ps-7 phylotypes on agricultural plants in Kosovo and the wastewater based environmental contamination needs to be further evaluated.

In the current study we were only able to monitor ARB in river and well water, but no physiochemical parameters. In a case study by Gashi et al.^107^ it was shown that the Llapi River water did not meet WHO drinking-water quality standards, as the analysis revealed that concentrations of Cr, Ni, Zn, Cu, and Fe were within WHO guideline limits at all sampling stations, while Cd and Pb exceeded allowable levels at all sites and Mn exceeded recommended limits at multiple locations. The Llapi River’s elevated Cd, Pb, and Mn concentrations may have a substantial impact on microbial ecology, encouraging the selection of bacteria that are resistant to metals and possibly antibiotics. The need for integrated antimicrobial and physicochemical monitoring is highlighted by the strong correlations between these metals, which point to common sources of contamination that may simultaneously impact chemical and microbiological water quality. Beside heavy metals the contamination with antimicrobial residues and biocidal compounds should be monitored in future studies to understand the AMR spread in more detail.

## Conclusions

Our study extended the knowledge on the environmental contamination of Kosovo with ARB and bacteria tolerant to biocidal compounds including potential pathogens. This is of specific importance because WHO estimates ranked Kosovo as the fourth-highest consumer of antibiotics among 13 non-EU Eastern European countries, with most human antibiotic use attributed to β-lactams, quinolones, macrolides, and sulfonamides^108^. Knowledge of AMR in Kosovo’s livestock sector is limited, but beta-lactams, sulfonamides, and tetracyclines are the most commonly used^36^. These strains from dairy animals were shown to contain priority AMR phenotypes including cephem, quinolone, and macrolide resistance.

Given the strong potential for co-selection of resistance to antibiotics, disinfectants, and heavy metals^101,102,109^, there is a clear need for extended surveillance of pollutants and AMR dispersal in water used for irrigation, irrigated soils, and produced agricultural products especially. A complete picture of the situation of AMR spread in the environment in Kosovo will be given by a coordinated investigation of pollutant dispersal, microbiological and molecular biological combined detection of AMR and detailed characterization of ARB. A recently published Antibiotic Resistance Awareness report for Kosovo illustrated that the public awareness of AMR in Kosovo is in general high, but the conceptual understanding of AMR is limited^110^. For a large part of the public the risk of AMR spread by contaminated irrigation water is likely not considered. We support the statement of the authors that “a multifaceted strategy including education, policy reforms, and international collaboration, is essential to mitigate AMR and preserve the efficacy of antibiotics for future generations”.

## Materials and methods

### Study Sites and Sampling Strategy

A systematically planned sampling campaign was performed in October 2021 in five geographically different vegetable-growing areas (locations 1 to 5, Fig. 1), in the region of Prishtina, the capital city of Kosovo, and Podujeva, a nearby town.

Water, soil, and vegetable samples were collected from privately managed agricultural fields with the permission of the landowners/farm managers. The plant material used in this study was collected exclusively from agricultural areas (cultivated fields) and did not involve wild populations of protected, endangered, or threatened species. No specific institutional or governmental permits were required under national regulations for soil and plant sampling in cultivated agricultural land. The position of each sample collection site was recorded using the Global Positioning System (GPS) application. The sampling locations were geographically dispersed, with great-circle distances ranging from approximately 1.2 km between location 3 and 4 to about 19 km between 1 and 2, while the remaining sites were separated by intermediate distances of 2–5 km. On the farm located at location 1, surface water from a nearby river was used for irrigation, while the farm at location 3 used a mixed irrigation system, both groundwater (well) and surface water (river) for irrigation purposes. The farms located at three different locations (locations 2, 4, and 5) were found to rely on groundwater from wells as an informal yet essential source of irrigation, highlighting the significance of this water source in the agricultural practices of this Kosovo region. The river at location 1 represents a site branch of the Llapi River, which was sampled upstream at location 3. The river sampling point at location 1 did not have any houses nearby and no clear signs of contamination were obvious. In contrast, at location 3 many houses were present in the surrounding area and a clear sign of contamination during the sampling campaign was observed. The water had a noticeable fecal odor.

For each location, irrigation water (river or well water), soil samples (upper soil layer, 0–20 cm depth) from agricultural fields planted with pepper, and the raw bell pepper fruits at the time of harvest were collected as follows.

Water samples were collected in 250- and 500-mL sterile glass bottles from the defined locations. The 250 mL water samples were sent to the Institute of Applied Microbiology, Justus-Liebig University in Giessen, Germany without any prior processing. The other 500 mL water samples were processed right after the sampling campaign in the Molecular Biology Laboratory of the University of Prishtina in Kosovo by collecting bacteria on sterile 0.22 μm membrane filters (Toyo Roshi Kaisha, Ltd., Japan). The membrane filters were placed in small-size Petri dishes with an immediate addition of 9 mL of autoclaved peptone water (PW, 7251, Liofilmchen, Italy) and then carefully sealed with parafilm (Avantor, VWR, USA) and kept cooled (4–8 °C). Upon transportation to Germany (under cooled conditions, 4–8 °C), both, the membrane filters and PW were transferred to sterile 50 mL screw cap tubes (Greiner Bio-One GmbH, Germany) for further processing.

Five soil samples from five locations (agricultural fields), each weighing approximately 200 g, were gathered by pooling 40 g of soil from five different spots within a field. Additionally, fresh, intact, and healthy pepper fruits were collected, with a total of five fruits (each weighing approximately 125 g) per location, obtained from the same spots within the same fields as soil samples. The samples were placed in individual sterile sample bags (Avantor).

All collected samples, including both, the unprocessed 250 mL water samples and filtered water samples, soil and pepper samples were immediately stored at 4–8 °C after collection. This cooling temperature was maintained consistently while the samples were stored in Kosovo and transported by plane to Germany (< 24 h), where they were further processed.

### Determination of pH and dry weights

The potential hydrogen (pH) of the water samples was assessed using a Mettler-Toledo measurement instrument (Schwerzenbach, Switzerland) upon arrival in Germany.

For dry weight determination of soil, 10 g of each soil sample was weighed and placed in an aluminum dish. The samples were then subjected to drying in an oven (MAGV GmbH, Londorf, Germany) at 105–110 °C for a period of 23 h. Subsequently, the samples were transferred to a Duran vacuum desiccator (Carl Roth, Karlsruhe, Germany) for an additional hour to reach equilibrium, after which they were weighed. Once each weight was documented, the samples were returned to the oven for further drying for up to 4 days. After this extended drying period, the samples were re-weighted, and their new weights were documented. The resulting dry weights of the soil samples were used after the bacterial cultivation for the calculation of colony-forming units (CFU) per g dry weight.

### Direct plating cultivation

For the direct plating cultivation, 100 µL of the cell suspensions were plated per agar plate using the spread plate method. This cultivation strategy enabled the quantification of culturable bacteria by counting colony-forming units (CFU) per mL of water or per g of soil or pepper samples. For water samples, undiluted water and 10- and 100-fold dilutions in autoclaved sodium chloride solution (NaCl, 0.9% w/v) were plated. Bacterial cells from soil and pepper fruit samples were detached from the sample material through mechanical treatment. For this purpose, 10 g of soil was placed with 90 mL of 0.22 μm filter-sterilized tetrasodium pyrophosphate buffer (TSPP, 0.2% w/v) into sterile strainer stomacher bags (Seward, Worthing, United Kingdom), which were mechanically treated in a Stomacher^®^ 400 circulator (Seward) at high speed (230 beats s^− 1^) for 60 s. For pepper fruit samples, five fruits collected from each location were diced into pieces using a sterilized scalpel and tweezers and mixed. Approximately 25 g of this mixed sample was placed with 150 mL autoclaved buffered peptone water (BPW; Carl Roth) into the stomacher bags and treated, respectively. The obtained cell suspensions were transferred to sterile 250 mL sterile glass bottles (SCHOTT) and used for plating. In addition, a ten-fold dilution was generated from the cell suspensions using autoclaved 0.9% NaCl and plated accordingly.

For the cultivation of total *E. coli* and 3GCR *E. coli*, the samples were plated on Tryptone Bile X-glucuronide agar (TBX; Carl Roth) and TBX supplemented with 1 mg cefotaxime L^− 1^ (CTX; Tokyo Chemicals Industry, Tokyo, Japan), respectively. All TBX plates were incubated under oxic conditions for 3 h at 37 °C, followed by a 21 h incubation at 44 °C. *E. coli* form green-blue colonies based on the β-D-glucuronidase activity. Media and incubation conditions were selected according to the standard operation procedure of the JPI-AMR project Antimicrobial Resistance Manure Intervention Strategies (ARMIS) (https://www.jpiamr.eu/projects/armis/).

Heterotrophic bacteria were cultivated on Mueller-Hinton agar (MH, Carl Roth) which was incubated at 37 °C under oxic conditions for 48 h. The MH agar was supplemented with 50 and 100 µg ^´^BAC-C12 mL^− 1^ (Tokyo Chemicals Industry, Tokyo, Japan) to culture the fraction of QAC tolerant heterotrophic bacteria. The BAC-C12 concentrations were selected based on the study of Heyde et al.^2^.

Colonies were counted, and the concentration of target bacteria in the original samples was given as CFU per mL of water, per g dry weight of soil, or per g fresh weight of pepper fruit. The limit of detection by this direct plating cultivation was 1 CFU per 100 µL water sample, 90 CFU per g soil, and 60 CFU per g pepper fruits.

### Filter-based direct cultivation

For water samples, bacterial cells from 10 mL water samples were collected on sterile 0.45 μm cellulose nitrate membrane filters (Hahnemühle Fine Art GmbH, Dassel, Germany), which were placed at the center of Petri dishes containing TBX agar. TBX plates were incubated as described above. Subsequently, the quantification of CFUs per mL in the water samples was determined as described above. The limit of detection was thereby 1 CFU per 10 mL water sample.

### Non-selective PE cultivation

In the case of low abundance of active target bacteria, the detection efficiency was enhanced by using a non-selective pre-enrichment cultivation strategy which allowed a qualitative (presence/absence) detection. All types of samples were subjected to non-selective pre-enrichment cultivation. For water samples transported to Germany, 5 mL of each sample was added to sterile 50-mL falcon tubes containing 20 mL of buffered peptone water (BPW, Carl Roth). In parallel, the 25 500 mL water samples filtered and preserved in peptone water (PW, ISO 7251, Liofilmchen) in Kosovo were directly used for pre-enrichment cultivation. For soil samples, 1 g of soil was added into 20 mL BPW, whereas for pepper fruit samples, the remaining cell suspensions detached from sliced pepper fruits in BPW were directly used for PE cultivation.

All PEs were incubated overnight at 37 °C on a horizontal shaker at 120 rpm (continuous shaking). Following the overnight incubation, 10 µL of the enrichment cultures were streaked by singulation streaking onto the same culture media as previously described for direct plating cultivation. The PE cultivation increased the limit of detection to 1 cell per 500 mL water, 1 g soil, and 25 g pepper fruit biomass.

### Characterization of cultivated bacteria

For the characterization of *E. coli* and 3GCR *E. coli* strains, up to eight green-blue colonies were selected per sample from TBX and TBX-CTX agar plates. For some samples, beige colonies were selected in parallel to identify the grown non-target bacteria. For the characterization of BAC-C12-tolerant bacteria, approximately three beige-colored colonies were taken per sample from the highest positive dilutions or enrichment streaks from MH-BAC-C12 agar plates. Singulation streaking based on single colonies was repeatedly performed until pure bacterial cultures were obtained. For strains from TBX, MH was used for subsequent cultivation, and for strains from TBX-CTX, MH with 1 mg CTX L^− 1^. All agar plates were incubated at 37 °C for 24 to 48 h.

For long-term preservation of pure bacterial cultures, two loops of fresh bacterial biomass were suspended twice in 500 µL of Gibco newborn calf serum (NBCS, ThermoFisher Scientific) and stored in 1.4 mL U-bottom push-cap tubes (Micronic, Netherlands) at -20 °C and − 80 °C. In parallel, one loop of fresh biomass was suspended in 500 µL of molecular-grade DNase- and RNase-free water (Carl Roth) to generate cell lysates for molecular characterization. Cell suspensions were subjected to three cycles of freezing (-20 °C) and heating (115 °C, 1 min, heating block). The reaction tubes were thoroughly mixed by vortexing after each heating step. Cell lysates were stored at -20 °C. Before PCR analysis, cell lysates were thawed, mixed, centrifuged, and stored on ice. The supernatant was used as a DNA template.

### Strain-level differentiation of bacterial strains by genomic DNA fingerprinting

Molecular genomic fingerprinting was performed to differentiate strains at the strain level (genotyping). The fingerprinting was carried out using BOX-PCR with the primer BOXA1R (5′-CTACGGCAAGGCGACGCTGACG-3′)^111^, as previously described^45^. The fingerprint patterns were analyzed in BioNumerics version 8 (Applied Maths, Belgium), with the unweighted pair group method and arithmetic average (UPGMA) clustering based on a similarity matrix generated with the Pearson correlation coefficient. Each unique fingerprint pattern was assigned as a distinct genotype.

### Diagnostic *E. coli* confirmation and *E. coli* phylotyping

A diagnostic PCR was used to confirm the identification of *E. coli* strains (blue-green colonies on TBX agar). The PCR amplified two gene variants, *gadA* and *gadB*, which encode the enzyme glutamate decarboxylase (GAD) gene, a specific indicator for the species *E. coli*^42,112^ using PCR conditions as described previously^113^.

### Phylogenetic identification of non-E. coli strains

Based on the strain differentiation by genomic DNA fingerprinting, representatives of all different genotypes were phylogenetically identified by partial 16S rRNA gene sequencing as described previously^47^. Primer systems EUB9F (5´-GAG TTT GAT CMT GGC TCA G-3′) and EUB1492R (5′- ACG GYT ACC TTG TTA CGA CTT-3′)^114^ were used to amplify the 16S rRNA gene according to Schauss et al.^45^. PCR products were sequenced by the Sanger method using primer EUB9F by LGC Genomics (Berlin, Germany). The obtained DNA sequences were manually corrected based on the electropherograms using MEGA11^115^. For phylogenetic classification, the Basic Local Alignment Search Tool (BLAST) version blastn blast+ v2.13.0 of the NCBI (https://blast.ncbi.nlm.nih.gov/) was used to determine sequence similarities to the next related type strains by using the curated 16S ribosomal RNA type strain database (updated as of 2023/01/12). The genus-level assignment was performed if strains shared a partial 16S rRNA gene sequence similarity of at least > 99.0% to type strain sequences representing species of the respective genus. This was the case for all studied strains.

For subsequent comparative analysis, the 16S rRNA gene sequences of the strains were added to the all species living tree project (LTP) type strain ARB database as described previously^116^. Based on the clustering in the phylogenetic tree and pairwise sequence similarities, strains were assigned to phylotypes.

### Multiplex-PCR-based screening for ESBL genes in ESBL *E. coli* strains

The presence of the three commonly abundant ESBL genes mainly detected in *Enterobacteriaceae*, *bla*_CTX−M_, *bla*_TEM_, and *bla*_SHV_ coding for CTX-M, TEM, and SHV ß-lactamases, was evaluated according to Schauss et al.^45^ using the multiplex-PCR scheme established by Monstein et al.^117^.

### Antibiotic susceptibility testing (AST) of 3GCR *E. coli* strains

Antibiotic susceptibility testing of 3GCR *E. coli* was performed by the broth microdilution method using the MRGN MicronautS System (96-well panel; Merlin, Bornheim-Hersel) as described by Müller et al.^118^. Strains were overnight cultivated on blood agar at 37 °C, suspended in autoclaved 0.9% NaCl solution to a McFarland standard 0.5 turbidity, and used for the inoculation of MH broth. According to the manufacturer’s description, 100 µL of the inoculated broth was used per well of the panel. Panels were sealed by plastic sheets and overnight incubated at 37 °C. Based on the visual observation of bacterial growth the MIC was determined for all antibiotics and the clinical breakpoint values provided by EUCAST Version 13.0 (European Committee on Antimicrobial Susceptibility Testing; https://www.eucast.org/clinical_breakpoints) database were used to define if *E. coli* strains were resistant (R) or sensitive (S) against the tested antibiotics.

The following antibiotics were tested: amikacin (AMK), cefotaxime (CTX), ceftazidime (CAZ), ceftazidime/3-aminophenylboronic acid (CZB), ceftazidime/avibactam (CAA), ceftolozan/tazobactam (CTA), chloramphenicol (CMP), ciprofloxacin (CIP), colistin (COL), fosfomycin (FOS), imipenem (IMP), levofloxacin (LEV), meropenem (MER), meropenem/3-aminophenylboronic acid (MEB) (KPC inhibitor), meropenem/ethylenediaminetetraacetic acid (MEE) (metallo-β-lactamases inhibitor), piperacillin (PIP), piperacillin/tazobactam (PIT), temocillin (TMO), tigecycline (TGC), and trimethoprim/sulfamethoxazole (T/S).

The multi-resistance status of the strains was evaluated using the MAR index. The MAR index was calculated as the ratio of the number of antibiotics to which a strain is resistant to the total number of antibiotics to which the strain was exposed according to Krumperman^74^. Here antibiotics were assigned to ten antibiotic classes, which were used as MAR index categories. As soon as one antibiotic per antibiotic class showed a resistance, the antibiotic class was counted as resistant. Based on EUCAST clinical breakpoints, MIC values were automatically converted to SR categories (Susceptible, Resistant) in BioNumerics Version 8 and were used to construct a dendrogram based on hierarchical clustering using the UPGMA as described previously^48^.

### QAC tolerance testing of 3GCR *E. coli* strains

Minimum inhibitory concentrations were determined for three QACs, BAC-C12, didecyldimethylammonium chloride (DADMAC-C10), and hexadecyltrimethylammonium chloride (ATMAC-C16), using a broth microdilution assay in accordance with the Clinical and Laboratory Standards Institute guidelines (M100-ED30)^119^ as described previously^2,47^. The following concentrations were tested: 0, 3.125, 6.25, 12.5, 25, 50, 100, and 200 µg BAC-C12 mL^− 1^; 0, 0.3125, 0.625, 1.25, 2.5, 5, 10, and 20 µg DADMAC-C10 mL^− 1^; and 0, 1.5625, 3.125, 6.25, 12.5, 25, 50, and 100 µg ATMAC-C16 mL^− 1^.

Cluster analysis of BOX-PCR based genomic fingerprint patterns of 3GCR *E. coli* strains by UPGMA clustering based on the Pearson correlation coefficient (BioNumerics, Applied Maths). Banding patterns were cropped in BioNumerics from different parts of one gel (See Supplementary Fig. 6. Strains with identical fingerprint pattern were assigned to the same genotype. Isolation source, genotype number, presence of potential ESBL genes (*bla*_CTX−M_, *bla*_TEM_, *bla*_SHV_) are given as subsequent information. Strain names include information of origin (KOS, Kosovo), month and year of sampling (1021, October 2021), sample type (W, water), the agar media used for cultivation (TC, TBX agar supplemented with CTX), and the chosen cultivation method (D, direct plating; P, pre-enrichment). Numeric values in front and behind the letter “W” are employed to denote specific location and sample replicate. “ND” not detected. Asterisks mark: Strains selected for AST; results are given in Fig. 4.

## Supplementary Information

Below is the link to the electronic supplementary material.

Supplementary Material 1

## Data Availability

All 16 S rRNA gene sequences obtained for bacterial strains were deposited into the primary database of the International Nucleotide Sequence Database Collaboration (INSDC) under the following Gen-Bank/EMBL/DDBJ accession numbers OQ918330-OQ918437.
