# Supplementary material for: Third generation cephalosporin-resistant (3GCR) Escherichia coli and biocide-tolerant heterotrophic bacteria in irrigation water used in Capsicum annuum cultivation areas in Kosovo
Source: Sci Rep. 2026 Apr 6;16:11615. doi: 10.1038/s41598-026-42583-z (PMC13056910; doi:10.1038/s41598-026-42583-z)
Supplement: Supplementary file 1 — Supplementary Material 1 [file 41598_2026_42583_MOESM1_ESM.pdf]

Supplementary Figures
to

Third generation cephalosporin-resistant (3GCR) *E. coli* and biocide-tolerant bacteria in irrigation water used in *Capsicum annuum* cultivation areas in Kosovo

Elona Tahiri Vela[1,2], Rreze M. Gecaj[2], Dipen Pulami[1], Arben Mehmeti[3], Peter Kämpfer[1], Stefanie P. Glaeser[1*]

[1]Institute of Applied Microbiology, Justus-Liebig-University Giessen, Giessen, Germany

[2]Department of Food Technology and Biotechnology, University of Prishtina, Prishtina, Kosovo

[3]Department of Plant Protection, University of Prishtina, Prishtina, Kosovo

*Correspondence

Stefanie.Glaeser@umwelt.uni-giessen.de

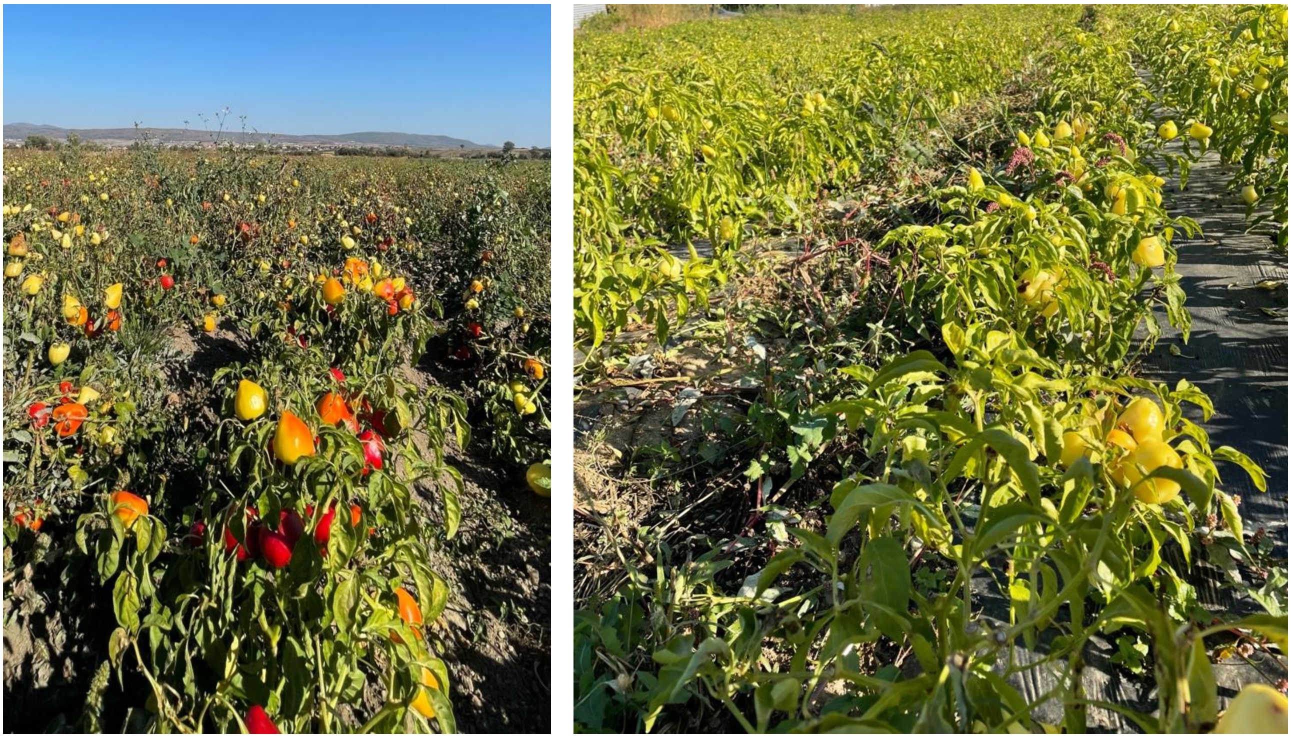

Supplementary Fig. S1 ***Capsicum annuum*** **(pepper) fields in Kosovo at the time of sampling.**

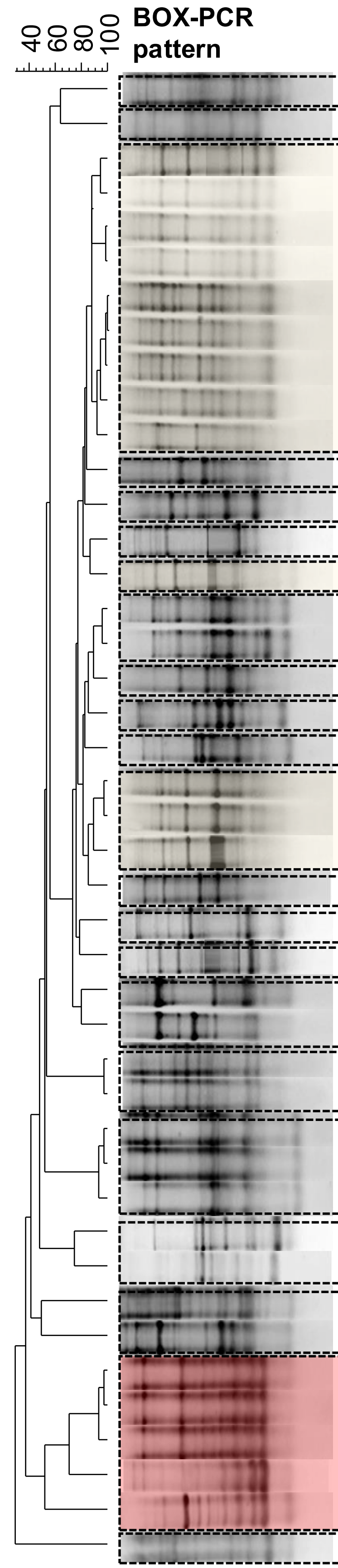

Strain	Isolation source	Location	Genotype	Phylotype
KOS_1021-3WPriver-TC-D1	River water	3	31	Ps15
KOS_1021-P2P-TC-P3	Pepper	2	5	Ps4
KOS_1021-P5P-MHB100-D1	Pepper	5	9	**Ps7**
KOS_1021-P5P-MHB100-P3	Pepper	5	9	**(Ps7)**
KOS_1021-P5P-MHB100-P1	Pepper	5	9	**Ps7**
KOS_1021-P5P-MHB100-P2	Pepper	5	9	**(Ps7)**
KOS_1021-P3P-MHB100-P1	Pepper	3	10	**Ps7**
KOS_1021-P3P-MHB100-P2	Pepper	3	10	**(Ps7)**
KOS_1021-P3P-MHB100-P3	Pepper	3	10	**(Ps7)**
KOS_1021-P3P-MHB100-P4	Pepper	3	11	**Ps7**
KOS_1021-P2P-MHB100-P3	Pepper	2	12	**Ps7**
KOS_1021-S3P-MHB100-D2	Soil	3	6	Ps5
KOS_1021-1WP-MHB100-D1	River water	1	2	Ps2
KOS_1021-P3P-MHB50-D1	Pepper	3	1	Ps1
KOS_1021-P4P-MHB100-D1	Pepper	4	13	**Ps7**
KOS_1021-S3P-MHB100-D1	Soil	3	8a	Ps6
KOS_1021-S3P-MHB100-D3	Soil	3	8b	(Ps6)
KOS_1021-S5P-MHB100-D1	Soil	5	7	Ps5
KOS_1021-4WP-MHB50-D1	Well water	4	22	Ps11
KOS_1021-S4P-MHB50-D2	Soil	4	24	Ps12
KOS_1021-P2P-MHB100-P1	Pepper	2	14	**Ps7**
KOS_1021-P2P-MHB100-P2	Pepper	2	14	**(Ps7)**
KOS_1021-P2P-MHB100-D1	Pepper	2	15	**Ps7**
KOS_1021-2W9-MHB100-P2	Well water	2	16	Ps8
KOS_1021-P1P-MHB50-D1	Pepper	1	21	Ps10
KOS_1021-P5P-MHB50-D2	Pepper	5	30	Ps14
KOS_1021-S1P-MHB100-D2	Soil	1	20	Ps9b
KOS_1021-S5P-MHB100-D2	Soil	5	17	Ps9a
KOS_1021-3WP-TC-P2	Well water	3	3	Ps2
KOS_1021-3WP-TC-P3	Well water	3	3	(Ps2)
KOS_1021-4W16-TC-P8b	Well water	4	4	(Ps3)
KOS_1021-4WP-TC-D3	Well water	4	4	Ps3
KOS_1021-4WP-TC-D1	Well water	4	4	(Ps3)
KOS_1021-P1P-MHB100-D1	Pepper	1	25	Ps12
KOS_1021-P2P-T-D1	Pepper	2	26	Ps12
KOS_1021-S3P-TC-P1	Soil	3	18	Ps9a
KOS_1021-S4P-TC-P1	Soil	4	19	Ps9a
**KOS_1021-2W8-TC-P6**	**Well water**	**2**	**27**	**Ps13**
**KOS_1021-2W8-TC-P7**	**Well water**	**2**	**27**	**(Ps13)**
**KOS_1021-2W8-TC-P5**	**Well water**	**2**	**27**	**(Ps13)**
**KOS_1021-1W3-TC-P2**	**River water**	**1**	**28**	**Ps13**
**KOS_1021-S1P-TC-P1**	**Soil**	**1**	**29**	**Ps13**
KOS_1021-2WP-TC-P2	Well water	2	23	Ps11

P. koreensis/ P. iranensis cluster

***P. aeruginosa* cluster**

Supplementary Fig. S2 Genotypic diversity of cultivated *Pseudomonas* strains. Clustering based on BOX-PCR patterns and was performed with the Pearson correlation coefficient and unweighted pair group method and arithmetic average (UPGMA) clustering using BioNumerics (Applied Maths). Banding patterns were cropped in BioNumerics from different parts of one gel and different gels; all gels contained three times the same standard to ensure comparability. The original gels are presented in Supplementary Figures S6-S11. Genotype assignment based on BOX-PCR pattern, phylotype assignment based on 16S rRNA gene sequences (see Fig. 6). Media used for isolation of the strains are given in the strain names (TC: TBX with 1 µg CTX mL^{-1} and MHB50/100: MH with 50 or 100 µg BAC-C12 mL^{-1}).

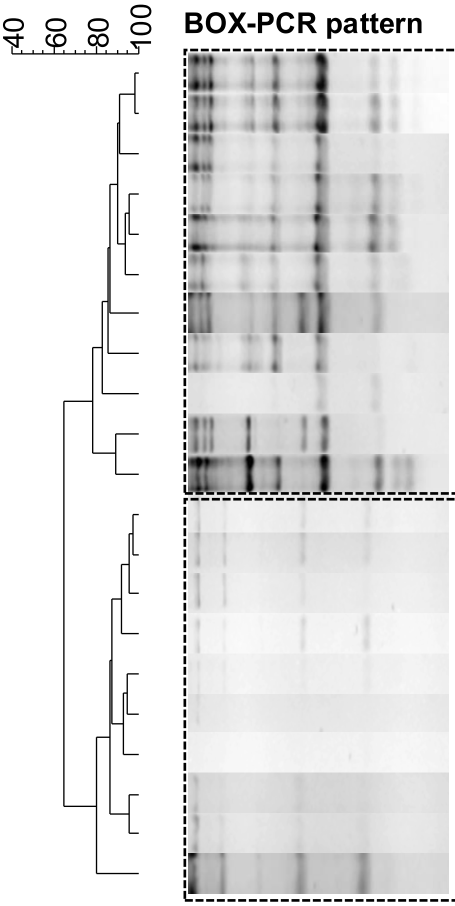

Strain	Isolation source	Location	Genotype	Phylotype
KOS_1021-1W4-TC-P1	River water	1	1	En1 *E. gallinarum/ E. casseliflavus* cluster
KOS_1021-1W5-TC-P1	River water	1	1	
KOS_1021-4W17-TC-P3	Well water	4	2	
KOS_1021-4W17-TC-P2	Well water	4	2	
KOS_1021-4W19-TC-P6	Well water	4	2	
KOS_1021-4W20-TC-P1	Well water	4	2	
KOS_1021-5W23-TC-P2a	Well water	5	3	
KOS_1021-4W18-TC-P3	Well water	4	4	
KOS_1021-4W19-TC-P5	Well water	4	N	
KOS_1021-S2P-TC-P2a	Soil	2	5	
KOS_1021-S2P-TC-P3b	Soil	2	6	
KOS_1021-3W13-TC-P1	Well water	3	7	En2 *E. faecium* cluster
KOS_1021-3W15-TC-P1	Well water	3	7	
KOS_1021-3W13-TC-P2	Well water	3	7	
KOS_1021-3W11-TC-P2	Well water	3	7	
KOS_1021-3W12-TC-P3	Well water	3	7	
KOS_1021-3W14-TC-P1	Well water	3	7	
KOS_1021-3W12-TC-P2	Well water	3	7	
KOS_1021-4W16-TC-P1	Well water	4	7	
KOS_1021-4W17-TC-P1	Well water	4	7	
KOS_1021-5W23-TC-P1	Well water	5	7	

N: no exact genotype asignment possible

Supplementary Fig. S3 Cluster analysis of cultivated *Enterococcus* spp. strains cultured as non-target bacteria on TBX with CTX. Clustering based on BOX-PCR patterns and was performed with the Pearson correlation coefficient and the UPGMA clustering using BioNumerics. Banding patterns were cropped in BioNumerics from different parts of one gel and different gels; all gels contained three times the same standard to ensure comparability. The original gels are presented in Supplementary Figures S10 and S11. Genotype assignment based on BOX-PCR pattern, phylotype assignment based on 16S rRNA gene sequences (see Fig. 6).

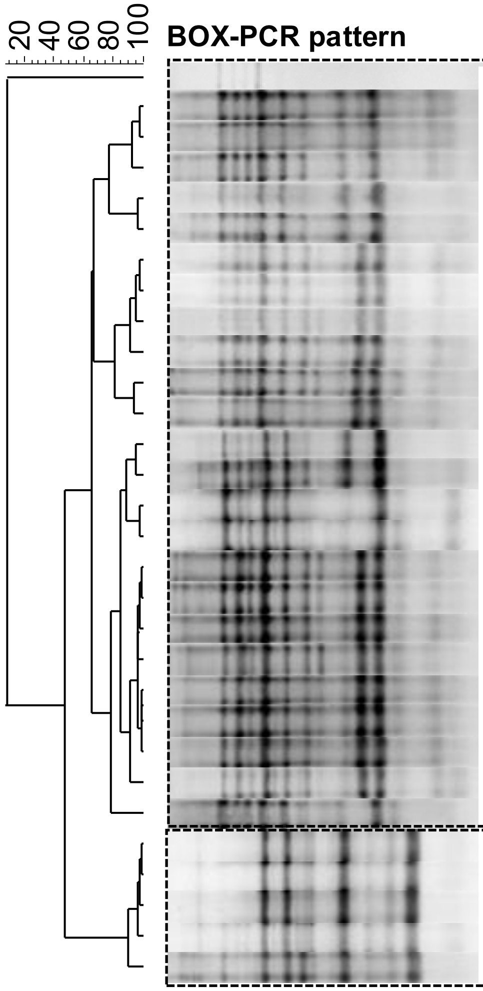

Strain	Isolation source	Location	Genotype	Phylotype
KOS_1021-1W5-MHB100-P3	River water	1	1	Mo1
KOS_1021-1W1-MHB100-P3b	River water	1	1	
KOS_1021-1W2-MHB100-P3	River water	1	1	
KOS_1021-1W1-MHB100-P3a	River water	1	1	
KOS_1021-2W7-MHB100-P1a	Well water	2	2	
KOS_1021-2W7-MHB100-P1b	Well water	2	2	
KOS_1021-2W7-MHB100-P3a	Well water	2	3	
KOS_1021-2W7-MHB100-P3b	Well water	2	3	
KOS_1021-2W7-MHB100-P2b	Well water	2	3	
KOS_1021-2W7-MHB100-P2a	Well water	2	3	
KOS_1021-2W6-MHB100-P1	Well water	2	3	
KOS_1021-2W6-MHB100-P2	Well water	2	3	
KOS_1021-2W8-MHB100-P2	Well water	2	4	
KOS_1021-2W8-MHB100-P3	Well water	2	4	
KOS_1021-5W22-MHB100-P2	Well water	5	5	
KOS_1021-5W23-MHB100-P1	Well water	5	5	
KOS_1021-2W10-MHB100-P2a	Well water	2	6	
KOS_1021-2W10-MHB100-P3a	Well water	2	6	
KOS_1021-2W10-MHB100-P2b	Well water	2	6	
KOS_1021-2W9-MHB100-P1	Well water	2	6	
KOS_1021-2W10-MHB100-P1a	Well water	2	6	
KOS_1021-2W10-MHB100-P1b	Well water	2	6	
KOS_1021-2W10-MHB100-P3b	Well water	2	6	
KOS_1021-2W8-MHB100-P1	Well water	2	6	
KOS_1021-5WP-MHB100-P1	Well water	5	7	
KOS_1021-5W23-MHB100-P2	Well water	5	8	Mo2
KOS_1021-5W25-MHB100-P1	Well water	5	8	
KOS_1021-5W25-MHB100-P3	Well water	5	8	
KOS_1021-5W24-MHB100-P2	Well water	5	8	
KOS_1021-4W18-MHB100-P3	Well water	4	9	

Supplementary Fig. S4 Cluster analysis of cultivated *Morganella* spp. strains tolerant to BAC-C12. Clustering based on BOX-PCR patterns and was performed with the Pearson correlation coefficient and the UPGMA clustering using BioNumerics. Banding patterns were cropped in BioNumerics from different parts of one gel and different gels; all gels contained three times the same standard to ensure comparability. The original gels are presented in Supplementary Figures S7 and S8. Genotype assignment based on BOX-PCR pattern, phylotype assignment based on 16S rRNA gene sequences (see Fig. 6).

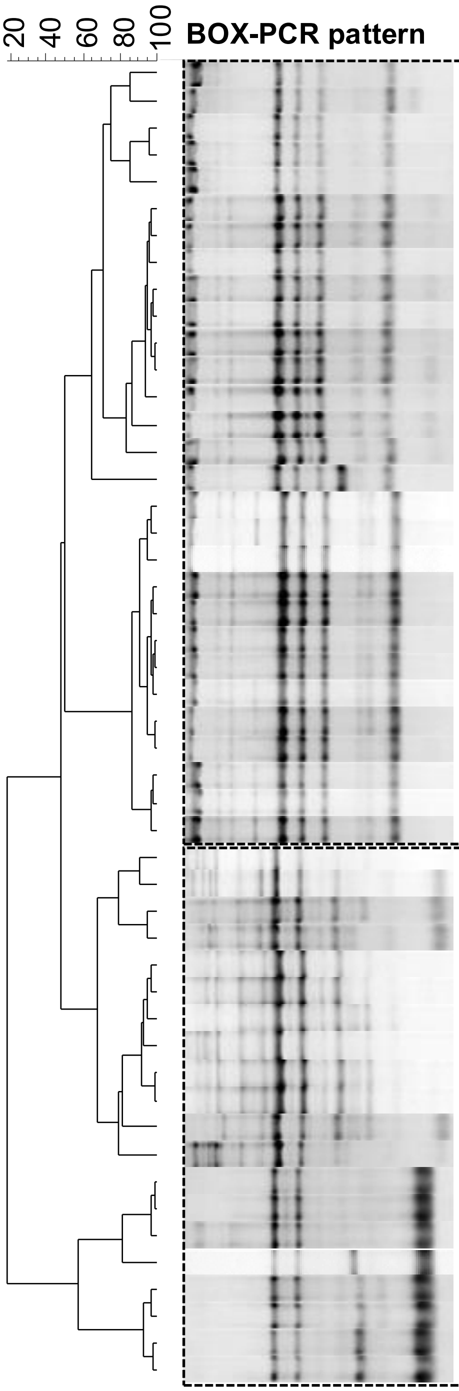

Strain	Isolation source	Location	Genotype	Phylotype
KOS_1021-4WP-MHB100-P1	Well water	4	1	
KOS_1021-4WP-MHB100-P2	Well water	4	1	
KOS_1021-1W4-MHB100-P3a	River water	1	1	
KOS_1021-1W5-MHB100-P1	River water	1	1	
**KOS_1021-1W5-MHB100-P2**	River water	1	1	
KOS_1021-1W1-MHB100-P1b	River water	1	1	
KOS_1021-1W2-MHB100-P2	River water	1	1	
KOS_1021-1W4-MHB100-P2a	River water	1	1	
KOS_1021-1W3-MHB100-P1a	River water	1	1	
KOS_1021-1W4-MHB100-P2b	River water	1	1	
KOS_1021-1W2-MHB100-P1	River water	1	1	
**KOS_1021-1W3-MHB100-P1b**	River water	1	1	
KOS_1021-1W3-MHB100-P3b	River water	1	1	
KOS_1021-1W3-MHB100-P3a	River water	1	1	
KOS_1021-1W1-MHB100-P1a	River water	1	1	Pr1
**KOS_1021-1W4-MHB100-P3b**	River water	1	2	
KOS_1021-4W20-MHB100-P2	Well water	4	3	
KOS_1021-4W20-MHB100-P3	Well water	4	3	
KOS_1021-4W20-MHB100-P1	Well water	4	3	
KOS_1021-4W16-MHB100-P2	Well water	4	3	
**KOS_1021-4W16-MHB100-P3**	Well water	4	3	
KOS_1021-4W18-MHB100-P1	Well water	4	3	
KOS_1021-4W18-MHB100-P2	Well water	4	3	
KOS_1021-4W19-MHB100-P2	Well water	4	3	
KOS_1021-4W17-MHB100-P1	Well water	4	3	
KOS_1021-4W17-MHB100-P3	Well water	4	3	
KOS_1021-4W19-MHB100-P1	Well water	4	3	
KOS_1021-4W19-MHB100-P3	Well water	4	3	
KOS_1021-4W16-MHB100-P1	Well water	4	3	
KOS_1021-5W24-MHB100-P3	Well water	5	4	
**KOS_1021-5W25-MHB100-P2**	Well water	5	5	
**KOS_1021-1W1-MHB100-P2**	River water	1	6	
**KOS_1021-2W6-MHB100-P3**	Well water	2	7	
KOS_1021-5W22-MHB100-P1	Well water	5	8	
KOS_1021-5W24-MHB100-P1	Well water	5	8	
**KOS_1021-5W23-MHB100-P3**	Well water	5	8	
KOS_1021-5W22-MHB100-P3	Well water	5	8	
KOS_1021-5W21-MHB100-P1	Well water	5	8	
KOS_1021-5W21-MHB100-P2	Well water	5	8	Pr2
KOS_1021-3W15-MHB100-P1	Well water	3	9	
KOS_1021-2W9-MHB100-P3	Well water	2	10	
KOS_1021-3W14-MHB100-P1	Well water	3	11	
**KOS_1021-4W17-MHB100-P2**	Well water	4	11	
KOS_1021-3W14-MHB100-P2	Well water	3	11	
**KOS_1021-5W21-MHB100-P3**	Well water	5	12	
**KOS_1021-1W3-MHB100-P2b**	River water	1	13	
KOS_1021-1W4-MHB100-P1b	River water	1	13	
**KOS_1021-1W3-MHB100-P2a**	River water	1	13	
KOS_1021-1W4-MHB100-P1a	River water	1	13	

Supplementary Fig. S5 Cluster analysis of cultivated *Providencia* spp. strains tolerant to BAC-C12.

Clustering based on BOX-PCR patterns and was performed with the Pearson correlation coefficient and the UPGMA clustering using BioNumerics. Banding patterns were cropped in BioNumerics from different parts of one gel and different gels; all gels contained three times the same standard to ensure comparability. The original gels are presented in Supplementary Figures S7 and S8. Genotype assignment based on BOX-PCR pattern, phylotype assignment based on 16S rRNA gene sequences (see Fig. 6).

Supplementary Figures 6 to 12

Original agarose gels used for BOX-PCR anaylsis

Lane	Sample
1	
2	**M 100bp**
3	KOS_1021-1W1-TC-P1
4	KOS_1021-1W1-TC-P2
5	KOS_1021-1W1-TC-P3
6	KOS_1021-1W1-TC-P4
7	KOS_1021-1W1-TC-P5
8	KOS_1021-1W1-TC-P6
9	KOS_1021-1W1-TC-P7
10	KOS_1021-1W1-TC-P8
11	KOS_1021-1W2-TC-P1
12	KOS_1021-1W2-TC-P2
13	KOS_1021-1W2-TC-P3
14	KOS_1021-1W2-TC-P4
15	KOS_1021-1W2-TC-P5
16	KOS_1021-1W2-TC-P6
17	KOS_1021-1W2-TC-P7
18	KOS_1021-1W2-TC-P8
19	**M 100bp**
20	KOS_1021-3WPriver-TC-D1
21	KOS_1021-3WPriver-TC-D2
22	KOS_1021-3WPriver-TC-D3
23	KOS_1021-3WPriver-TC-D4
24	KOS_1021-3WPriver-TC-D5
25	KOS_1021-3WPriver-TC-D6
26	KOS_1021-3WPriver-TC-D7
27	KOS_1021-3WPriver-TC-D8
28	KOS_1021-3WPriver-TC-P2b
29	KOS_1021-3WPriver-TC-P2c
30	KOS_1021-3WPriver-TC-P4
31	**M 100bp**

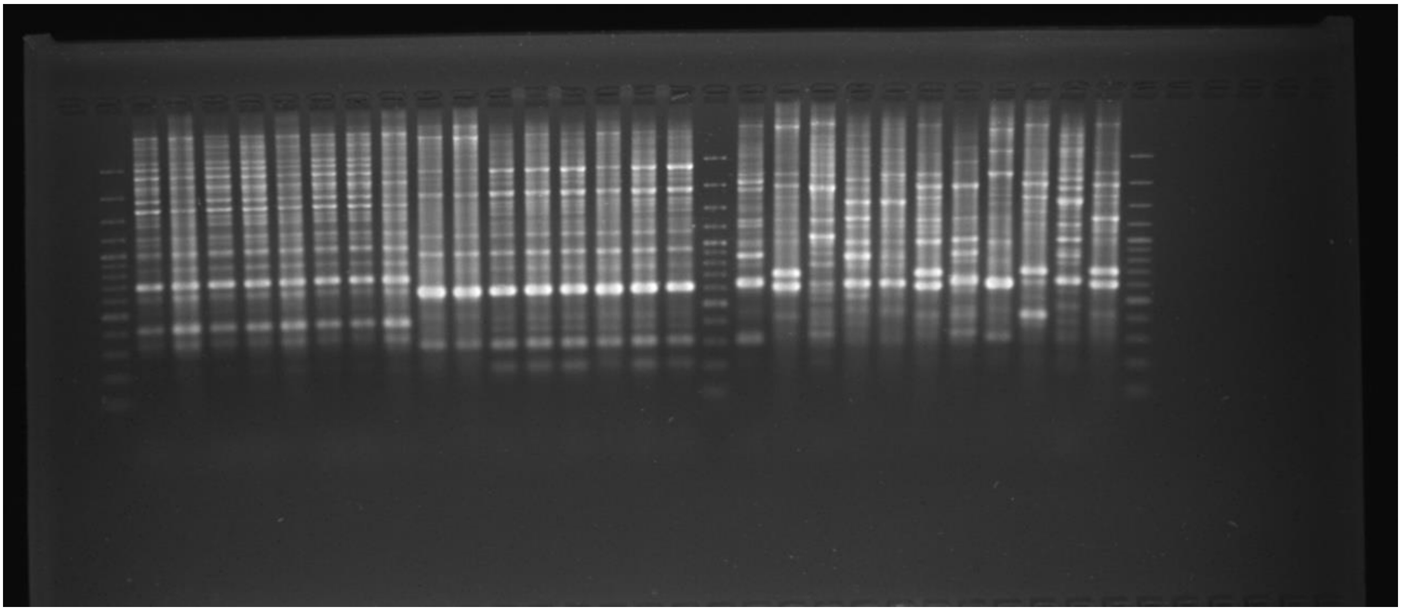

Lane	Sample
1	
2	**M 100bp**
3	KOS_1021-5W21-TC-P1
4	KOS_1021-5W21-TC-P2
5	KOS_1021-5W21-TC-P3
6	KOS_1021-5W22-TC-P1
7	KOS_1021-5W22-TC-P2
8	KOS_1021-5W22-TC-P3
9	KOS_1021-5W22-TC-P4
10	KOS_1021-5W22-TC-P5
11	KOS_1021-5W22-TC-P6
12	KOS_1021-5W22-TC-P7
13	KOS_1021-5W22-TC-P8
14	**M 100bp**
15	KOS_1021-5W24-TC-P1
16	KOS_1021-5W24-TC-P2
17	KOS_1021-5W24-TC-P3
18	KOS_1021-5W24-TC-P4
19	KOS_1021-5W24-TC-P5
20	KOS_1021-5W24-TC-P6
21	KOS_1021-5W24-TC-P7
22	KOS_1021-5W24-TC-P8
23	KOS_1021-5W25-TC-P1
24	KOS_1021-5W25-TC-P2
25	KOS_1021-5W25-TC-P3
26	NTC
27	**M 100bp**

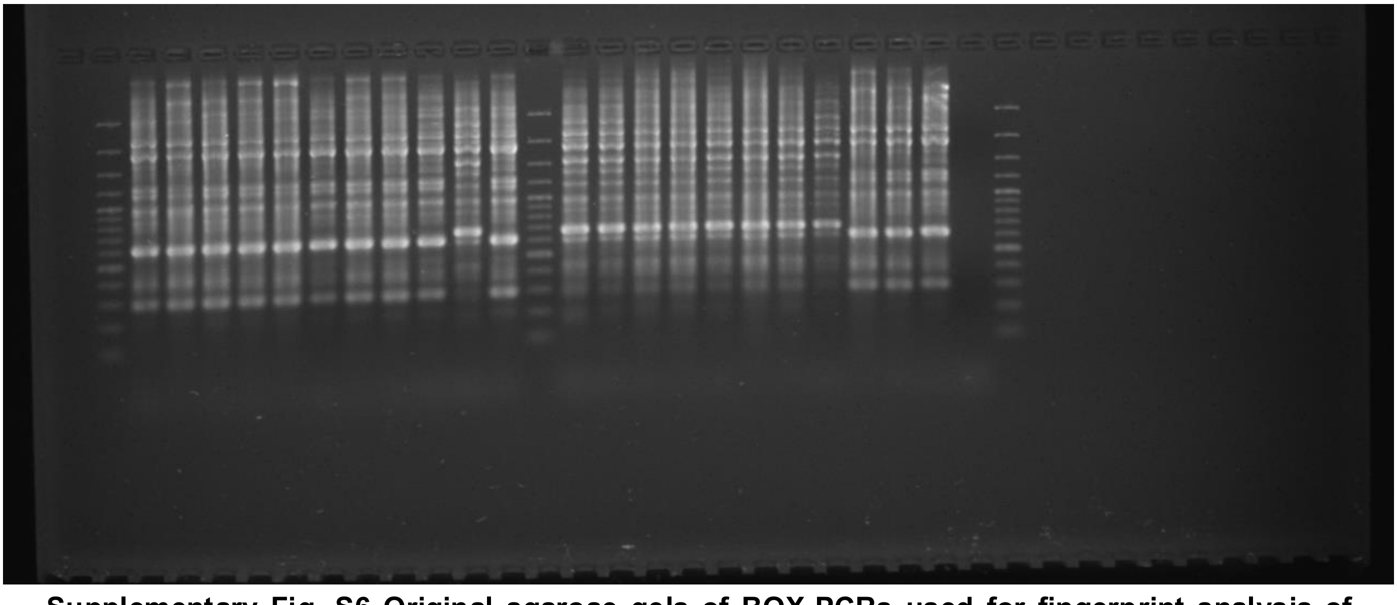

Supplementary Fig. S6 Original agarose gels of BOX-PCRs used for fingerprint analysis of bacterial strains in BioNumerics. BOX-PCR products were separated by agarose gel electrophoresis and visualized after ethidium bromide staining. The gels were used for analysis show in Figure 3. Lane M: 100 bp DNA Ladder Plus (ThermoScientific). All the other lanes correspond to strains analysed in this study.

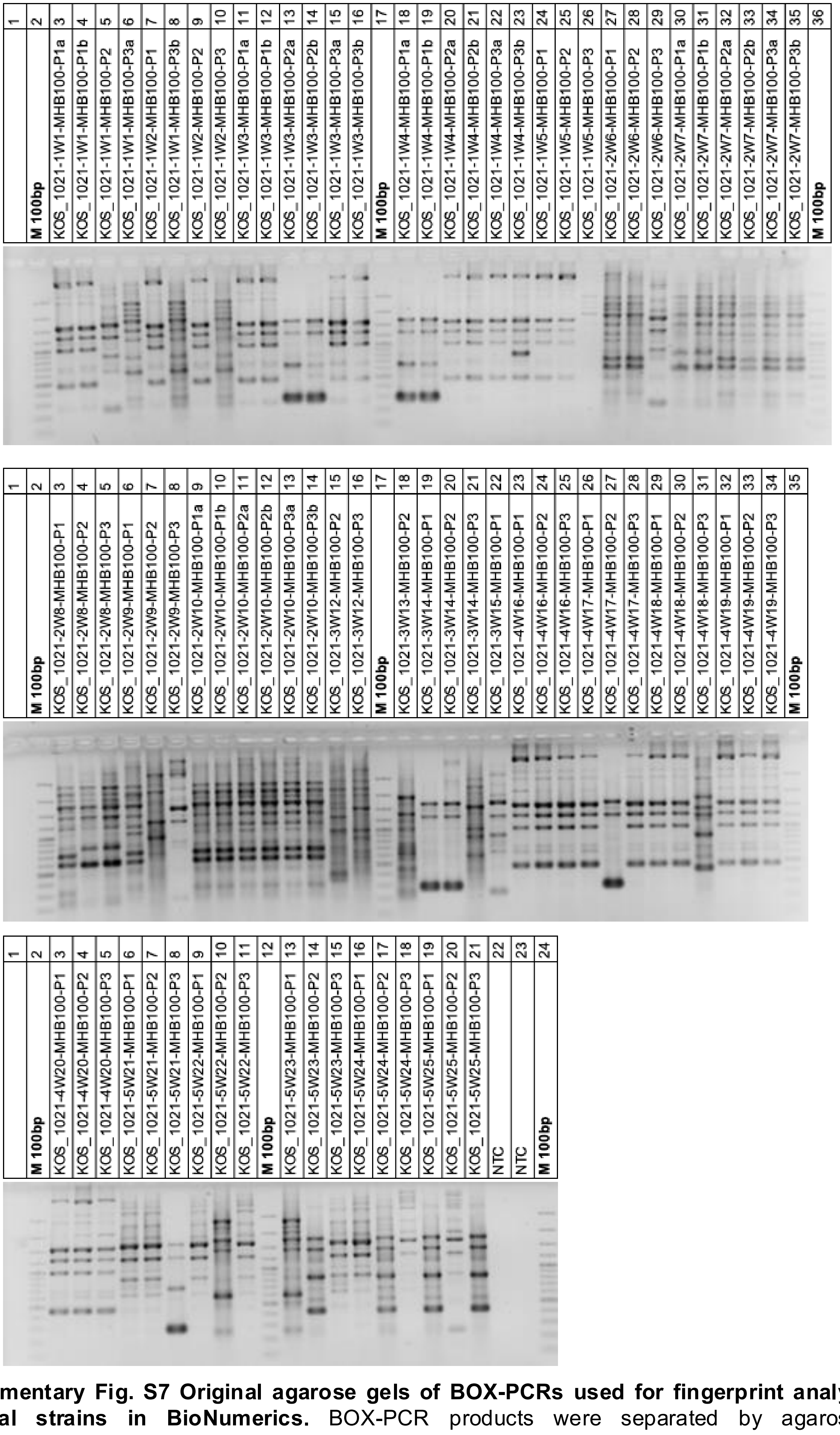

Supplementary Fig. S7 Original agarose gels of BOX-PCRs used for fingerprint analysis of bacterial strains in BioNumerics. BOX-PCR products were separated by agarose gel electrophoresis and visualized after ethidium bromide staining. Here inverted but unprocessed gels are presented. The gels were used for analysis show in Supplementary Figures S2, S4, and S5. Lane M: 100 bp DNA Ladder Plus (ThermoScientific). All the other lanes correspond to strains analysed in this study.

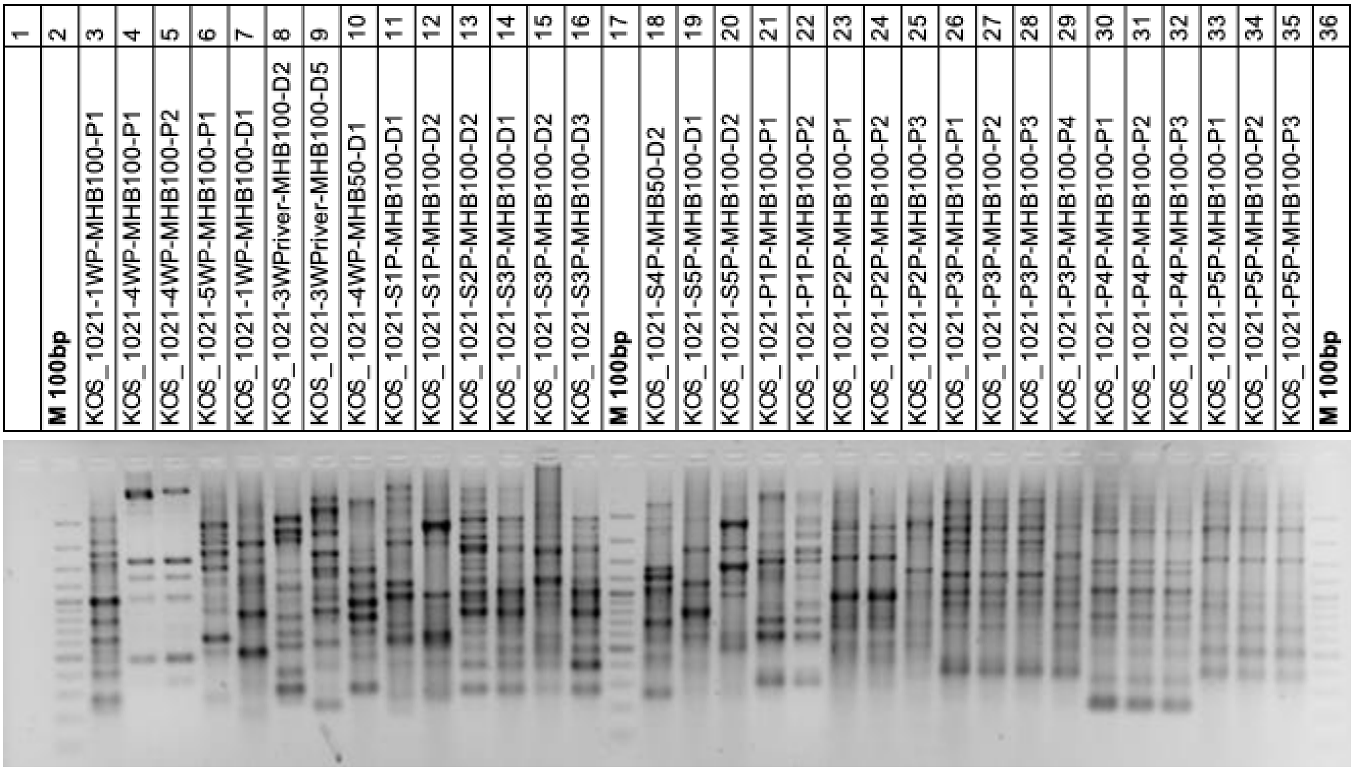

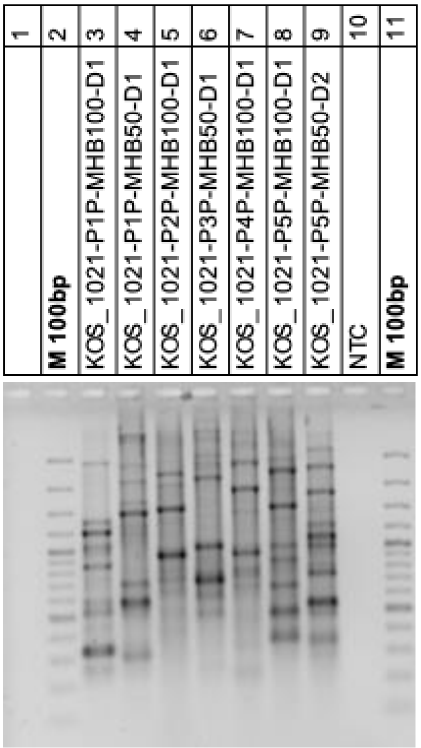

Supplementary Fig. S8 Original agarose gels of BOX-PCRs used for fingerprint analysis of bacterial strains in BioNumerics. BOX-PCR products were separated by agarose gel electrophoresis and visualized after ethidium bromide staining. Here inverted but unprocessed gels are presented. The gels were used for analysis show in Supplementary Figures S2, S4, and S5. Lane M: 100 bp DNA Ladder Plus (ThermoScientific). All the other lanes correspond to strains analysed in this study.

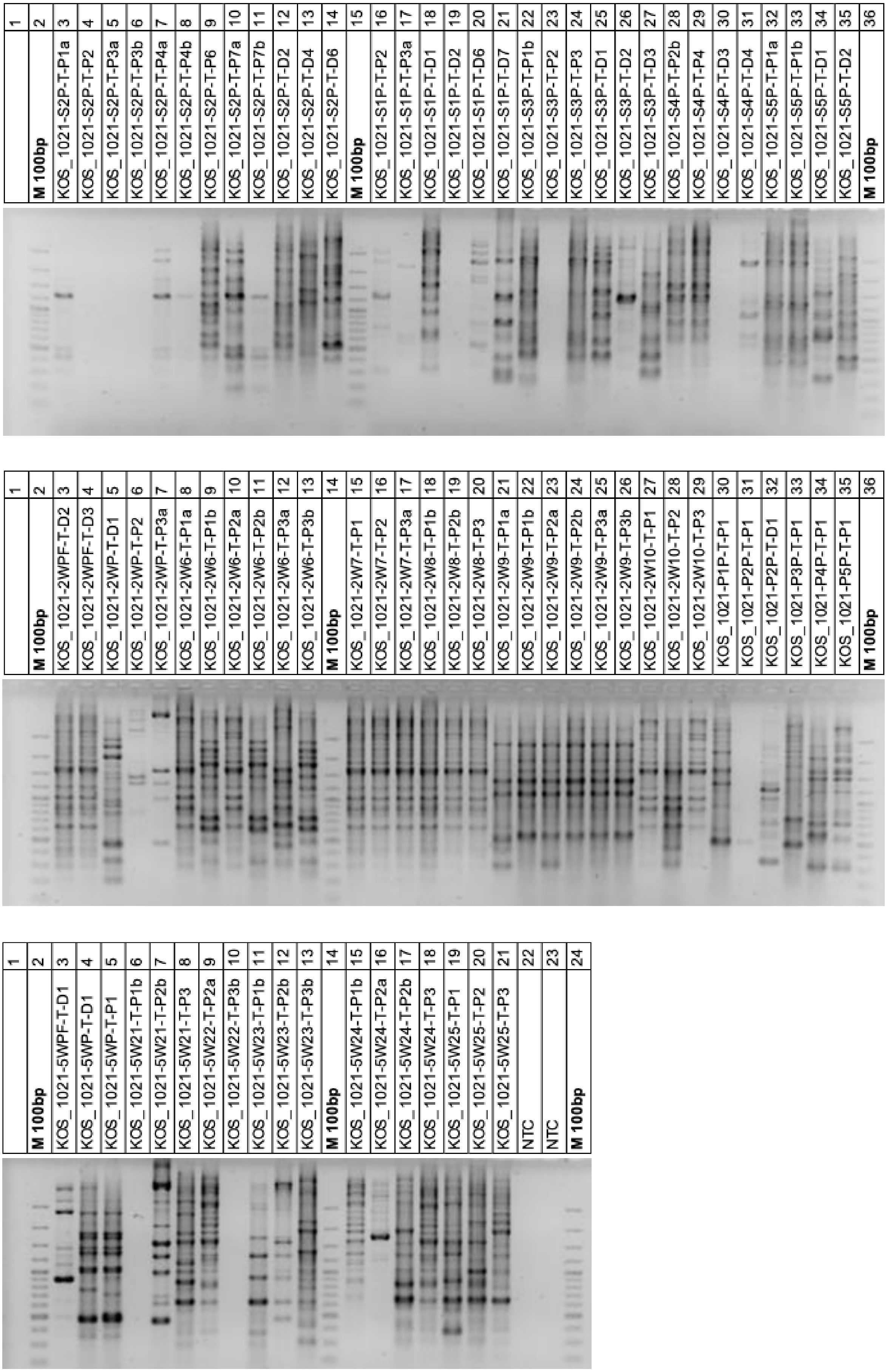

Supplementary Fig. S9 Original agarose gels of BOX-PCRs used for fingerprint analysis of bacterial strains in BioNumerics. BOX-PCR products were separated by agarose gel electrophoresis and visualized after ethidium bromide staining. Here inverted but unprocessed gels are presented. The gels were used for analysis show in Supplementary Figures S2. Lane M: 100 bp DNA Ladder Plus (ThermoScientific). All the other lanes correspond to strains analysed in this study.

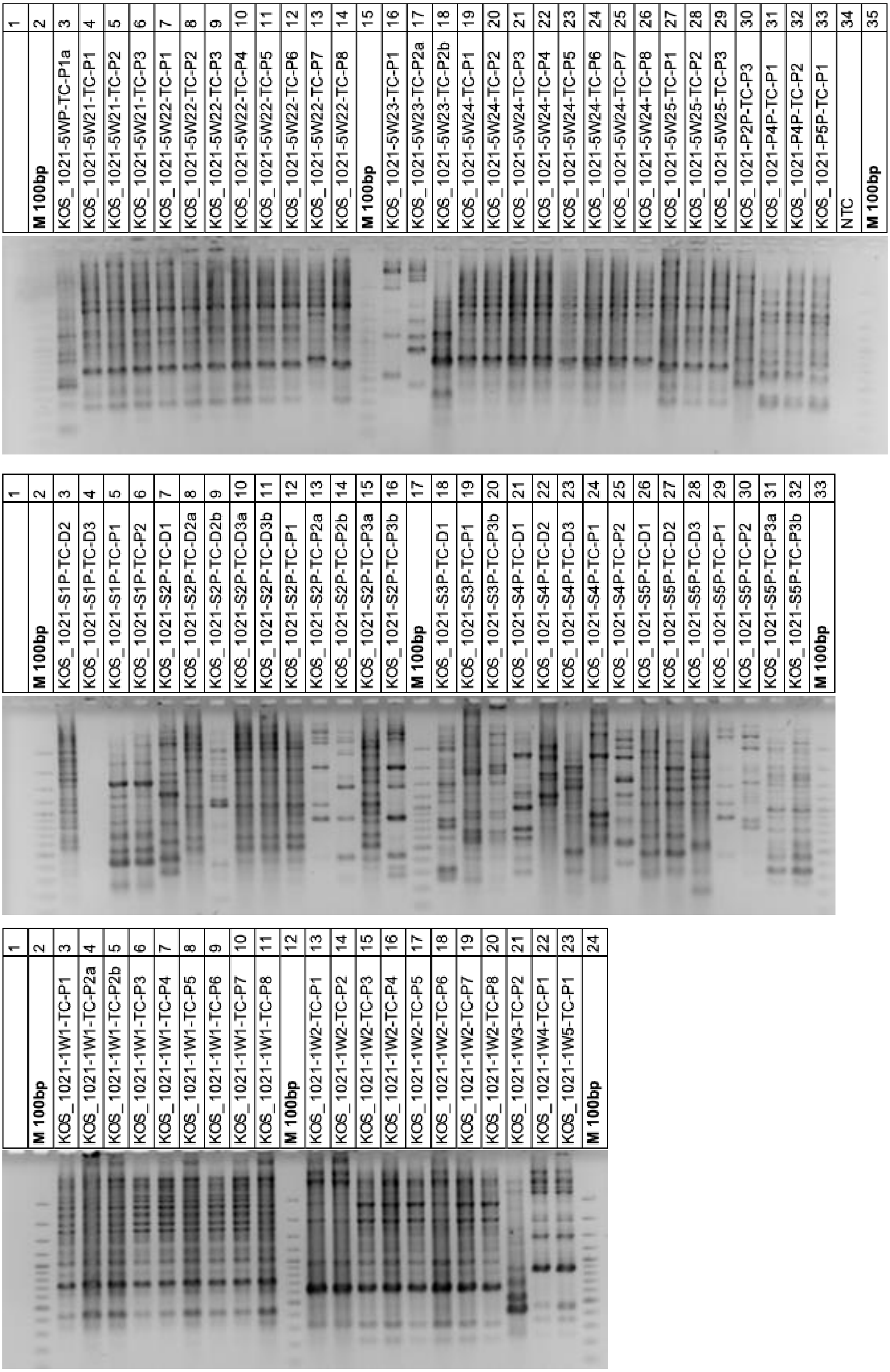

Supplementary Fig. S10 Original agarose gels of BOX-PCRs used for fingerprint analysis of bacterial strains in BioNumerics. BOX-PCR products were separated by agarose gel electrophoresis and visualized after ethidium bromide staining. Here inverted but unprocessed gels are presented. The gels were used for analysis show in Supplementary Figures S2. Lane M: 100 bp DNA Ladder Plus (ThermoScientific). All the other lanes correspond to strains analysed in this study.

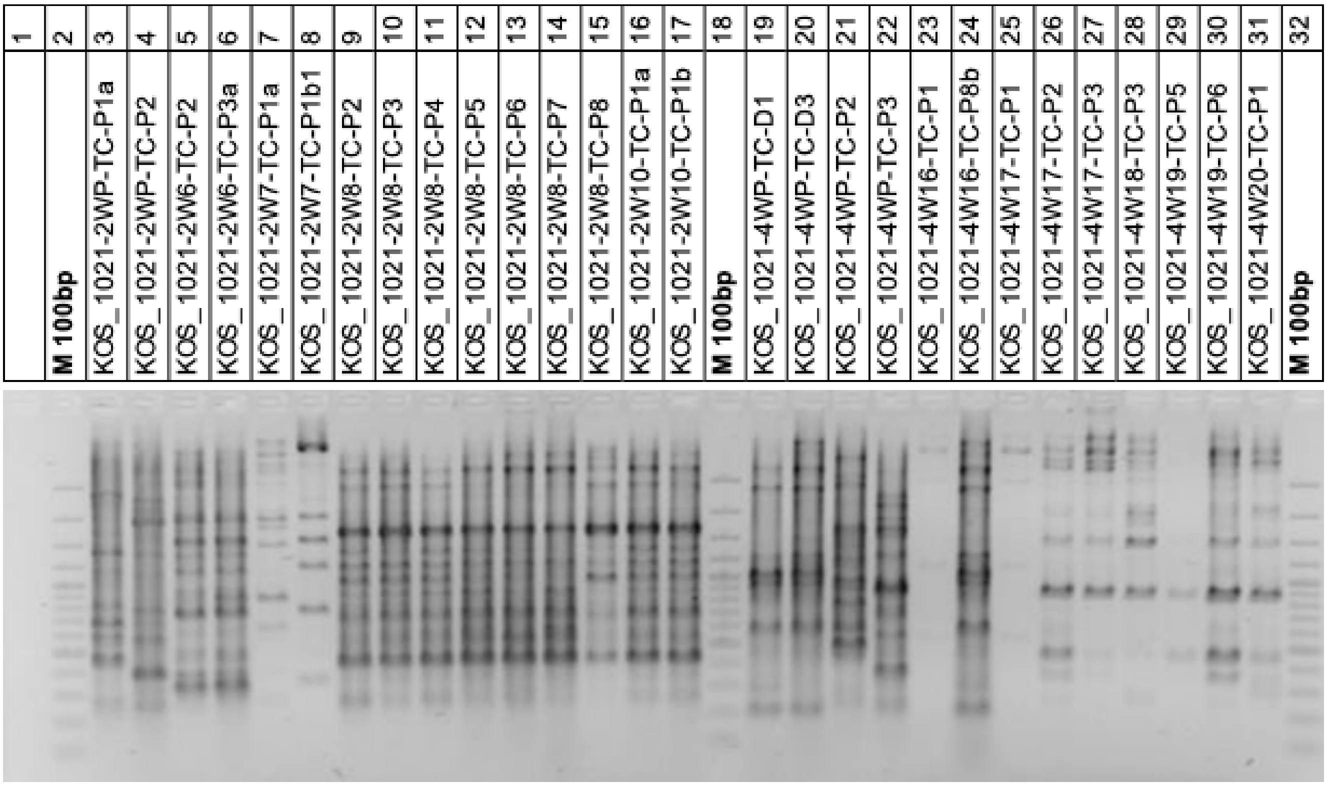

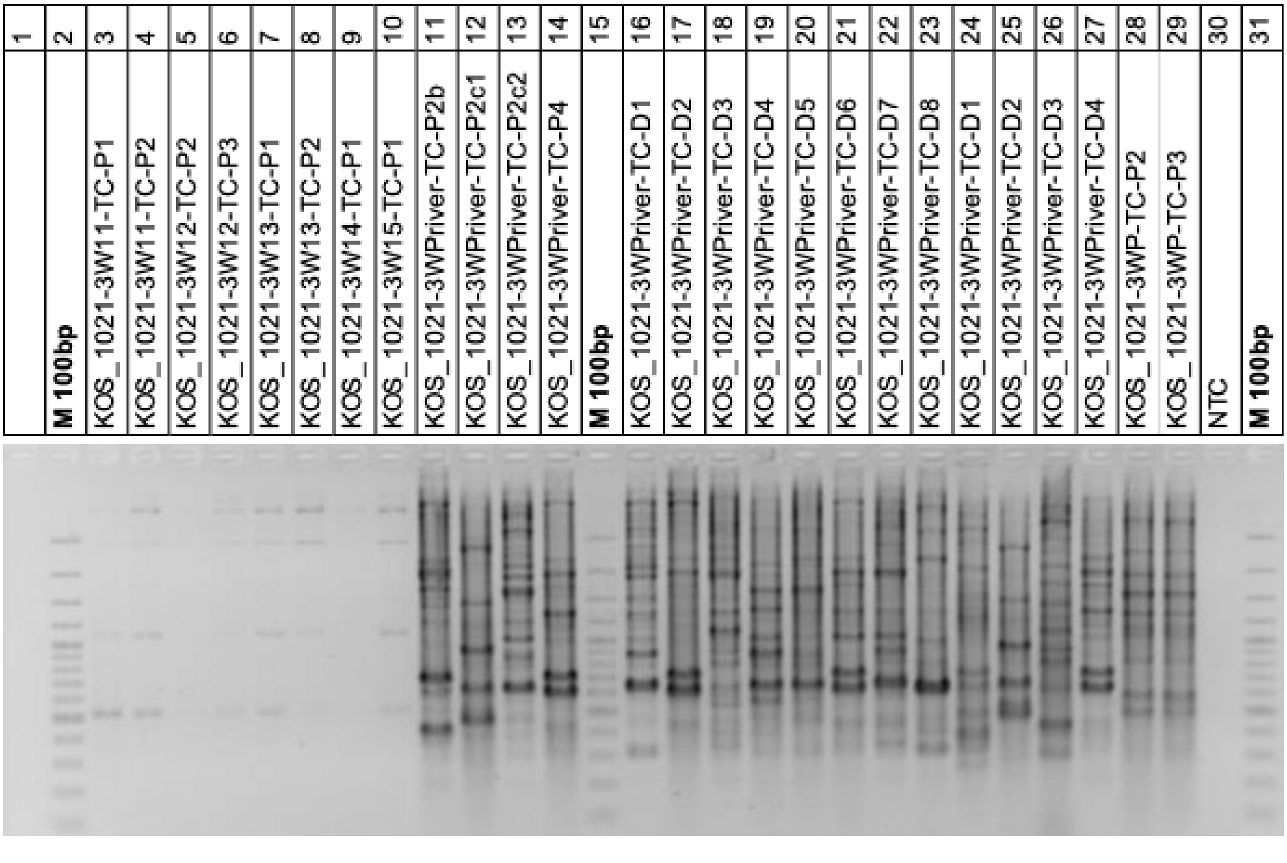

Supplementary Fig. S11 Original agarose gels of BOX-PCRs used for fingerprint analysis of bacterial strains in BioNumerics. BOX-PCR products were separated by agarose gel electrophoresis and visualized after ethidium bromide staining. Here inverted but unprocessed gels are presented. The gels were used for analysis show in Supplementary Figures S2 and S3. Lane M: 100 bp DNA Ladder Plus (ThermoScientific). All the other lanes correspond to strains analysed in this study.

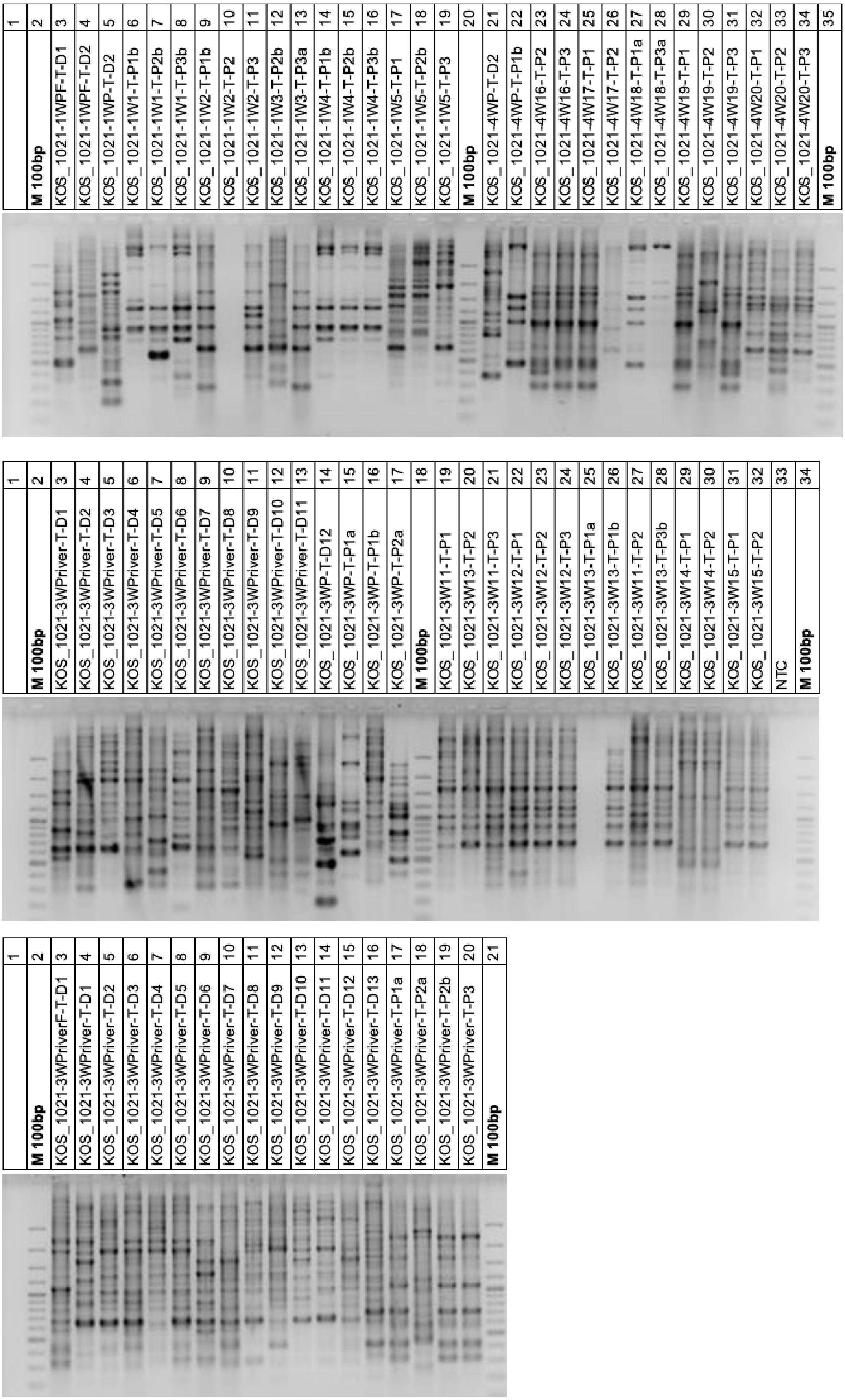

Supplementary Fig. S12 Original agarose gels of BOX-PCRs used for fingerprint analysis of bacterial strains in BioNumerics. BOX-PCR products were separated by agarose gel electrophoresis and visualized after ethidium bromide staining. Here inverted but unprocessed gels are presented. The gels were used for analysis show in Supplementary Figures S2, S4, and S5. Lane M: 100 bp DNA Ladder Plus (ThermoScientific). All the other lanes correspond to strains analysed in this study.
